# Supplementary material for: Mouse Vendor Influence on the Bacterial and Viral Gut Composition Exceeds the Effect of Diet
Source: Viruses. 2019 May 13;11(5):435. doi: 10.3390/v11050435 (PMC6563299; doi:10.3390/v11050435)
Supplement: Supplementary file 1 [file viruses-11-00435-s001.zip › Supplemental materials_revised/Supplemental materials/Rasmussen et al. 2019 - Viruses - SM_revised_13052019.docx]

**Table S1.** Illumina NextSeq sequencing details of both the 16S rRNA gene amplicons and metaviromes of faecal content isolated from both cecum and colon. Only reads used for vOTU’s are showed, reads used for contaminations are excluded. The table list the amount of b- and vOTUs after filtering based on 10x coverage and abundance as described in methods. BC = bacterial community, VC = viral community.

|  | **BC** | | **VC** | |
| --- | --- | --- | --- | --- |
|  | **Cecum** | **Colon** | **Cecum** | **Colon** |
| Mean sequencing depth (reads) | 318395 | 168388 | 829533 | 456452 |
| STD (reads) | 173421 | 28037 | 336144 | 110063 |
| Lowest sequencing depth (reads) | 47182 | 47004 | 212545 | 63183 |
| Maximum sequencing depth (reads) | 808971 | 223787 | 1621360 | 643913 |
| vOTUs before 10x coverage filtering | - | - | 15513 | 15513 |
| bOTU/vOTUs before abundance filtering | 6205 | 6205 | 12624 | 12624 |
| bOTU/vOTUs after abundance filtering | 2653 | 3259 | 3441 | 2073 |


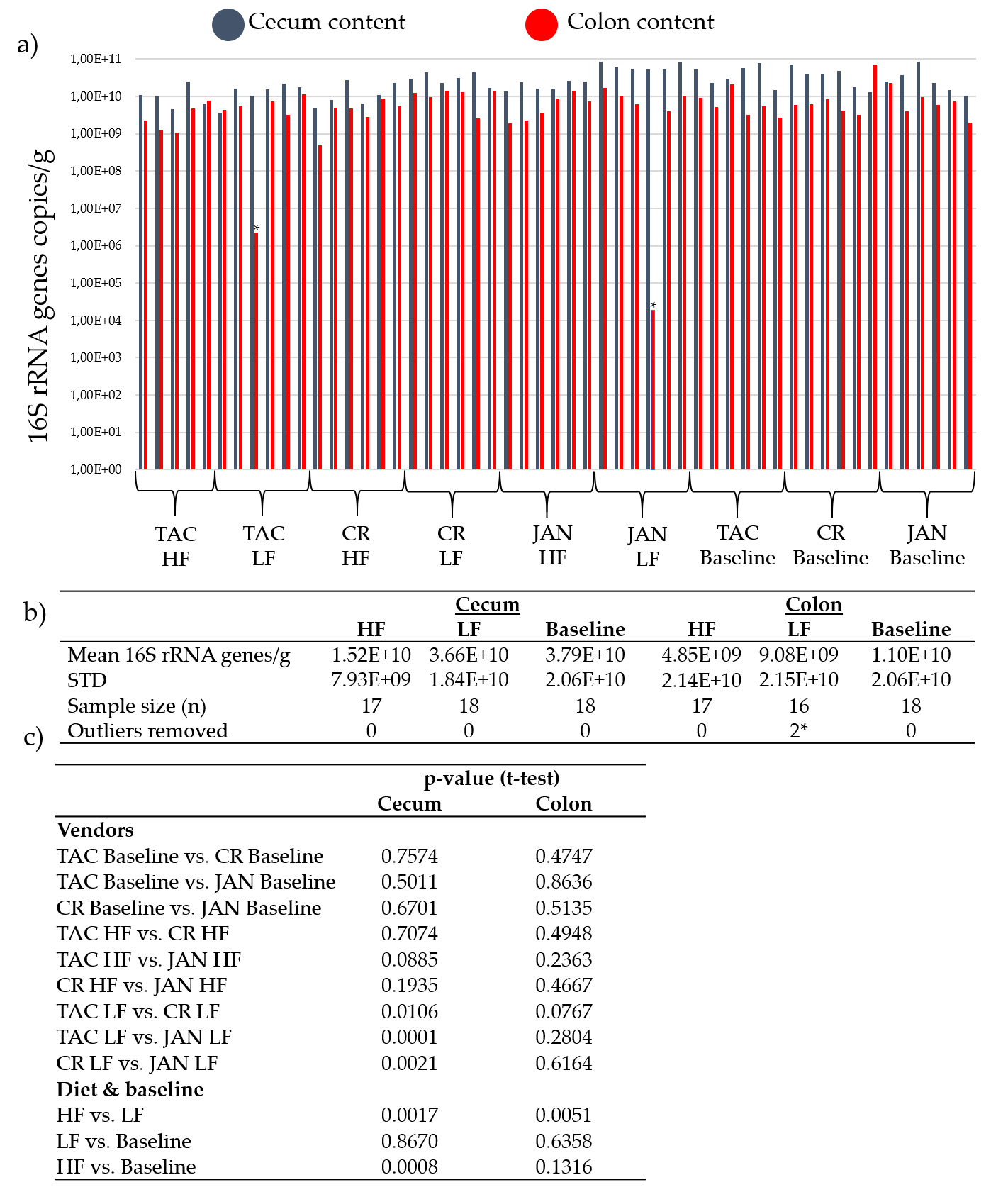


**Figure S1.** a) Mean qPCR results of universal primers targeting 16S rRNA genes (V3 region as described in methods) to estimate bacterial density in the included samples. All samples are included in the bar plot to show sample variations. b) Mean 16S rRNA gene copies/g in cecum and colon samples in the HF, LF and baseline groups. c) A t-test of differences in mean 16S rRNA gene copies/g between HF, LF, baseline, and between vendors. HF diet = high-fat, LF = low-fat diet, CR = Charles River, JAN = Janvier, TAC = Taconic.
* samples were removed as outliers.

## Figure S2: Bacterial and viral α-diversity analysis with other indices – Cecum

**
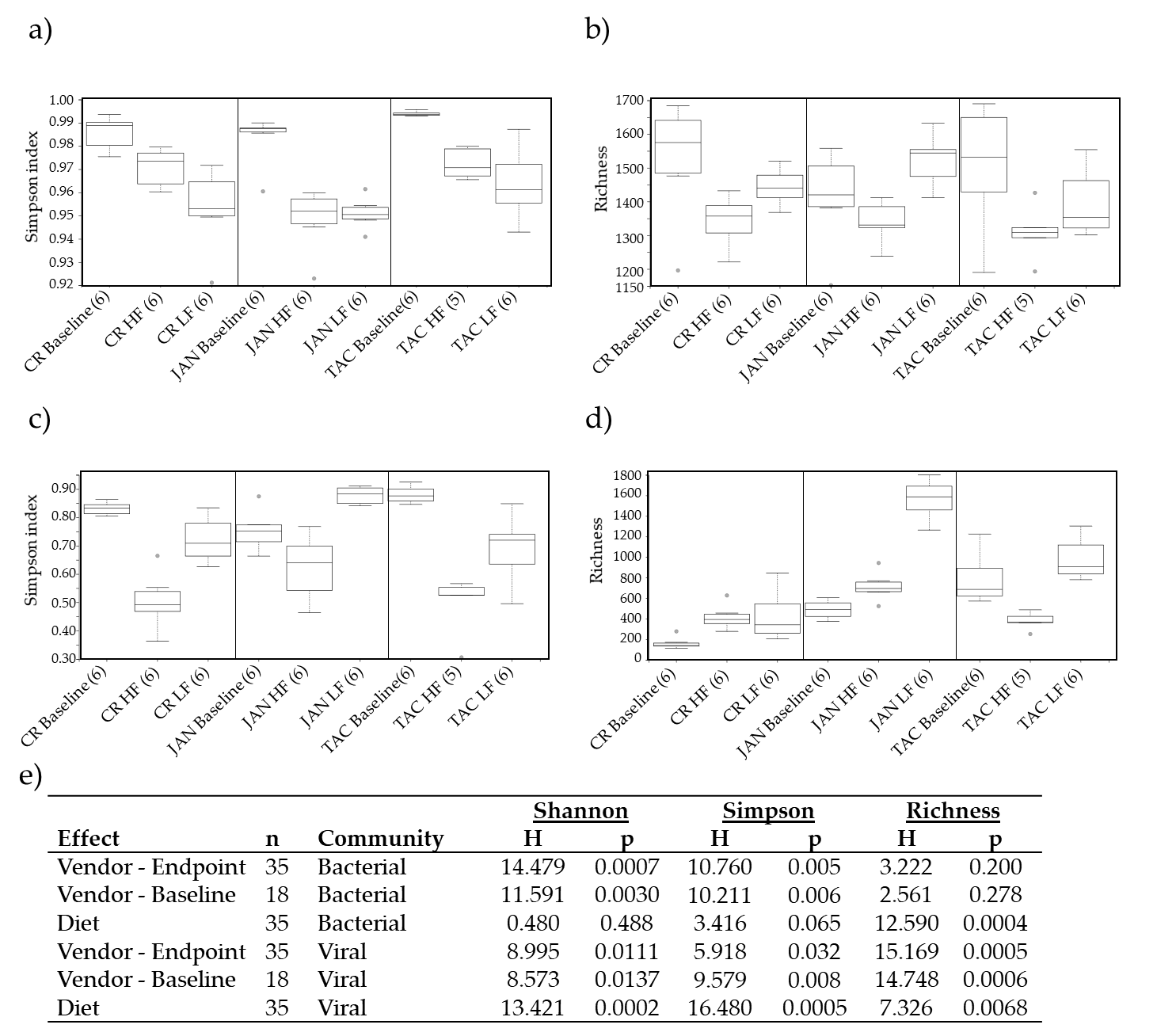
**

**Figure S2.** α-diversity (Simpson index and Richness) of the caecal bacterial community in a) and b), and viral community in c) and d). The parentheses show the number of samples from each group included in the plot and grey dots indicate outliers. e) Kruskal Wallis group analysis of the α-diversity indices of the effects of diet and vendor at baseline (5 weeks of age) and endpoint (18 weeks of age). LF = low-fat diet, HF = high-fat diet, CR = Charles River, JAN = Janvier, TAC = Taconic.

**
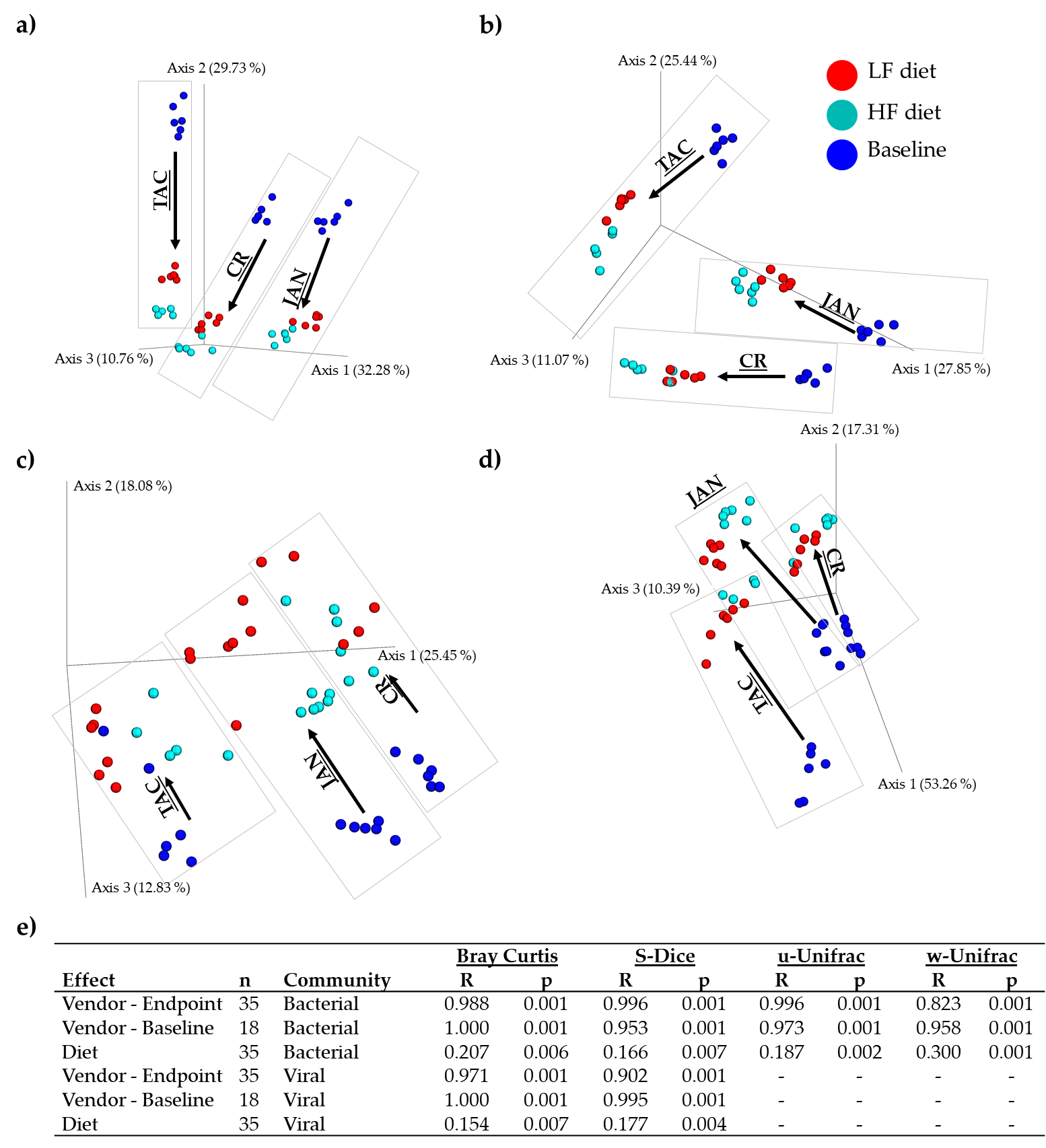
**

**Figure S3.** β-diversity (Sørensen-Dice, unweighted- and weighted unifrac metrics) of the caecal bacterial community in a), b), and d), and viral community in c) illustrated with PCoA plots. e) ANOSIM of the effects of diet and vendor at baseline (5 weeks of age) and endpoint (18 weeks of age). CR = Charles River, JAN = Janvier, TAC = Taconic. Gray boxes frame the samples associated to the mice vendor. S-Dice = Sørensen-Dice, u = unweighted, w = weighted.

**Table S1.** Pairwise Kruskal Wallis (H and p-values) comparison of α-diversity of both the caecal bacterial (BC) and viral community (VC) of all applied indices. BL = baseline, HF = high-fat diet, LF = low-fat diet.

|  |  |  | **Shannon-BC** | | **Shannon-VC** | | **Simpson-BC** | | **Simpson -VC** | | **Richness-BC** | | **Richness-VC** | |
| --- | --- | --- | --- | --- | --- | --- | --- | --- | --- | --- | --- | --- | --- | --- |
| **Gr. 1** | **Gr. 2** | **n** | **H** | **p** | **H** | **p** | **H** | **p** | **H** | **p** | **H** | **p** | **H** | **p** |
| CR_BL | CR_HF | 12 | 6.564 | 0.010 | 6.564 | 0.010 | 5.026 | 0.025 | 8.308 | 0.004 | 3.692 | 0.055 | 7.880 | 0.005 |
|  | CR_LF | 12 | 8.308 | 0.004 | 1.256 | 0.262 | 8.308 | 0.004 | 5.026 | 0.025 | 2.077 | 0.150 | 6.564 | 0.010 |
|  | JAN_BL | 12 | 1.641 | 0.200 | 0.000 | 1.000 | 0.923 | 0.337 | 3.692 | 0.055 | 2.077 | 0.150 | 8.308 | 0.004 |
|  | JAN_HF | 12 | 8.308 | 0.004 | 0.923 | 0.337 | 8.308 | 0.004 | 8.308 | 0.004 | 3.692 | 0.055 | 8.308 | 0.004 |
|  | JAN_LF | 12 | 8.308 | 0.004 | 8.308 | 0.004 | 8.308 | 0.004 | 5.026 | 0.025 | 0.410 | 0.522 | 8.308 | 0.004 |
|  | TAC_BL | 12 | 7.410 | 0.006 | 8.308 | 0.004 | 5.769 | 0.016 | 5.769 | 0.016 | 0.026 | 0.873 | 8.308 | 0.004 |
|  | TAC_HF | 11 | 7.500 | 0.006 | 7.500 | 0.006 | 4.033 | 0.045 | 7.500 | 0.006 | 4.033 | 0.045 | 6.533 | 0.011 |
|  | TAC_LF | 12 | 5.769 | 0.016 | 0.103 | 0.749 | 6.564 | 0.010 | 5.026 | 0.025 | 2.077 | 0.150 | 8.308 | 0.004 |
| CR_HF | CR_LF | 12 | 4.333 | 0.037 | 4.333 | 0.037 | 5.026 | 0.025 | 6.564 | 0.010 | 5.769 | 0.016 | 0.231 | 0.631 |
|  | JAN_BL | 12 | 5.026 | 0.025 | 6.564 | 0.010 | 4.333 | 0.037 | 7.410 | 0.006 | 1.641 | 0.200 | 1.256 | 0.262 |
|  | JAN_HF | 12 | 8.308 | 0.004 | 3.692 | 0.055 | 8.308 | 0.004 | 3.103 | 0.078 | 0.026 | 0.873 | 7.410 | 0.006 |
|  | JAN_LF | 12 | 6.564 | 0.010 | 8.308 | 0.004 | 6.564 | 0.010 | 8.308 | 0.004 | 7.410 | 0.006 | 8.308 | 0.004 |
|  | TAC_BL | 12 | 8.308 | 0.004 | 8.308 | 0.004 | 8.308 | 0.004 | 8.308 | 0.004 | 3.103 | 0.078 | 6.182 | 0.013 |
|  | TAC_HF | 11 | 0.533 | 0.465 | 0.033 | 0.855 | 0.133 | 0.715 | 0.300 | 0.584 | 0.533 | 0.465 | 0.133 | 0.715 |
|  | TAC_LF | 12 | 0.026 | 0.873 | 5.026 | 0.025 | 0.923 | 0.337 | 5.026 | 0.025 | 0.410 | 0.522 | 8.308 | 0.004 |
| CR_LF | JAN_BL | 12 | 7.410 | 0.006 | 0.641 | 0.423 | 6.564 | 0.010 | 0.641 | 0.423 | 0.006 | 0.936 | 0.923 | 0.337 |
|  | JAN_HF | 12 | 2.564 | 0.109 | 0.000 | 1.000 | 0.231 | 0.631 | 2.077 | 0.150 | 5.769 | 0.016 | 3.692 | 0.055 |
|  | JAN_LF | 12 | 0.923 | 0.337 | 8.308 | 0.004 | 0.641 | 0.423 | 8.308 | 0.004 | 3.692 | 0.055 | 8.308 | 0.004 |
|  | TAC_BL | 12 | 8.308 | 0.004 | 4.333 | 0.037 | 8.308 | 0.004 | 8.308 | 0.004 | 0.923 | 0.337 | 4.333 | 0.037 |
|  | TAC_HF | 11 | 2.700 | 0.100 | 7.500 | 0.006 | 3.333 | 0.068 | 7.500 | 0.006 | 5.633 | 0.018 | 0.033 | 0.855 |
|  | TAC_LF | 12 | 2.564 | 0.109 | 0.103 | 0.749 | 0.923 | 0.337 | 0.026 | 0.873 | 1.256 | 0.262 | 6.564 | 0.010 |
| JAN_BL | JAN_HF | 12 | 8.308 | 0.004 | 0.641 | 0.423 | 8.308 | 0.004 | 4.333 | 0.037 | 1.641 | 0.200 | 5.769 | 0.016 |
|  | JAN_LF | 12 | 8.308 | 0.004 | 7.410 | 0.006 | 7.410 | 0.006 | 5.769 | 0.016 | 2.564 | 0.109 | 8.308 | 0.004 |
|  | TAC_BL | 12 | 8.308 | 0.004 | 4.333 | 0.037 | 8.308 | 0.004 | 5.769 | 0.016 | 1.641 | 0.200 | 7.410 | 0.006 |
|  | TAC_HF | 11 | 5.633 | 0.018 | 7.500 | 0.006 | 3.333 | 0.068 | 7.500 | 0.006 | 2.133 | 0.144 | 3.333 | 0.068 |
|  | TAC_LF | 12 | 5.769 | 0.016 | 0.103 | 0.749 | 5.026 | 0.025 | 0.923 | 0.337 | 0.641 | 0.423 | 8.308 | 0.004 |
| JAN_HF | JAN_LF | 12 | 0.410 | 0.522 | 8.308 | 0.004 | 0.000 | 1.000 | 8.308 | 0.004 | 7.880 | 0.005 | 8.308 | 0.004 |
|  | TAC_BL | 12 | 8.308 | 0.004 | 4.333 | 0.037 | 8.308 | 0.004 | 8.308 | 0.004 | 3.103 | 0.078 | 0.000 | 1.000 |
|  | TAC_HF | 11 | 7.500 | 0.006 | 2.700 | 0.100 | 7.500 | 0.006 | 2.700 | 0.100 | 0.833 | 0.361 | 7.500 | 0.006 |
|  | TAC_LF | 12 | 5.769 | 0.016 | 0.231 | 0.631 | 2.077 | 0.150 | 1.256 | 0.262 | 0.410 | 0.522 | 5.026 | 0.025 |
| JAN_LF | TAC_BL | 12 | 8.308 | 0.004 | 6.564 | 0.010 | 8.308 | 0.004 | 0.026 | 0.873 | 0.026 | 0.873 | 8.308 | 0.004 |
|  | TAC_HF | 11 | 5.633 | 0.018 | 7.500 | 0.006 | 7.500 | 0.006 | 7.500 | 0.006 | 6.533 | 0.011 | 7.500 | 0.006 |
|  | TAC_LF | 12 | 5.026 | 0.025 | 6.564 | 0.010 | 3.103 | 0.078 | 6.564 | 0.010 | 3.692 | 0.055 | 7.410 | 0.006 |
| TAC_BL | TAC_HF | 11 | 7.500 | 0.006 | 7.500 | 0.006 | 7.500 | 0.006 | 7.500 | 0.006 | 2.700 | 0.100 | 7.500 | 0.006 |
|  | TAC_LF | 12 | 8.308 | 0.004 | 1.641 | 0.200 | 8.308 | 0.004 | 7.410 | 0.006 | 1.641 | 0.200 | 2.077 | 0.150 |
| TAC_HF | TAC_LF | 11 | 0.133 | 0.715 | 7.500 | 0.006 | 1.633 | 0.201 | 4.033 | 0.045 | 2.133 | 0.144 | 7.500 | 0.006 |

**Table S3.** Pairwise ANOSIM (R and p-values) comparison of β-diversity of both the caecal bacterial (BC) and viral community (VC) of all applied metrices. The R-value is an arbitrary level of separation. S-Dice = Sørensen-Dice, u = unweighted, w = weighted.

|  |  |  | **Bray-Curtis-BC** | | **Bray-Curtis-VC** | | **S-Dice-BC** | | **S-Dice-VC** | | **u-UniFrac-BC** | | **w-UniFrac-BC** | |
| --- | --- | --- | --- | --- | --- | --- | --- | --- | --- | --- | --- | --- | --- | --- |
| **Gr. 1** | **Gr. 2** | **n** | **R** | **p** | **R** | **p** | **R** | **p** | **R** | **p** | **R** | **p** | **R** | **p** |
| CR_BL | CR_HF | 12 | 1.000 | 0.003 | 1.000 | 0.003 | 1.000 | 0.005 | 0.937 | 0.001 | 1.000 | 0.003 | 1.000 | 0.001 |
|  | CR_LF | 12 | 1.000 | 0.005 | 1.000 | 0.001 | 1.000 | 0.003 | 0.970 | 0.002 | 1.000 | 0.004 | 1.000 | 0.004 |
|  | JAN_BL | 12 | 1.000 | 0.002 | 1.000 | 0.006 | 1.000 | 0.002 | 0.987 | 0.005 | 0.990 | 0.003 | 0.830 | 0.004 |
|  | JAN_HF | 12 | 1.000 | 0.001 | 1.000 | 0.005 | 1.000 | 0.006 | 0.996 | 0.005 | 1.000 | 0.002 | 1.000 | 0.003 |
|  | JAN_LF | 12 | 1.000 | 0.003 | 1.000 | 0.003 | 1.000 | 0.001 | 1.000 | 0.003 | 1.000 | 0.002 | 1.000 | 0.003 |
|  | TAC_BL | 12 | 1.000 | 0.001 | 1.000 | 0.002 | 1.000 | 0.002 | 1.000 | 0.001 | 1.000 | 0.004 | 1.000 | 0.003 |
|  | TAC_HF | 11 | 1.000 | 0.003 | 1.000 | 0.003 | 1.000 | 0.004 | 1.000 | 0.004 | 1.000 | 0.005 | 1.000 | 0.003 |
|  | TAC_LF | 12 | 1.000 | 0.002 | 1.000 | 0.003 | 1.000 | 0.002 | 1.000 | 0.006 | 1.000 | 0.002 | 1.000 | 0.006 |
| CR_HF | CR_LF | 12 | 0.980 | 0.002 | 0.652 | 0.004 | 0.850 | 0.003 | 0.344 | 0.031 | 0.850 | 0.001 | 0.650 | 0.003 |
|  | JAN_BL | 12 | 1.000 | 0.001 | 1.000 | 0.006 | 1.000 | 0.004 | 1.000 | 0.006 | 1.000 | 0.002 | 1.000 | 0.002 |
|  | JAN_HF | 12 | 1.000 | 0.001 | 1.000 | 0.002 | 1.000 | 0.003 | 0.985 | 0.002 | 1.000 | 0.004 | 0.970 | 0.003 |
|  | JAN_LF | 12 | 1.000 | 0.002 | 1.000 | 0.001 | 1.000 | 0.002 | 0.989 | 0.004 | 1.000 | 0.005 | 1.000 | 0.002 |
|  | TAC_BL | 12 | 1.000 | 0.003 | 1.000 | 0.003 | 1.000 | 0.002 | 1.000 | 0.005 | 1.000 | 0.003 | 1.000 | 0.002 |
|  | TAC_HF | 11 | 0.980 | 0.001 | 1.000 | 0.004 | 1.000 | 0.004 | 1.000 | 0.001 | 1.000 | 0.004 | 0.800 | 0.001 |
|  | TAC_LF | 12 | 1.000 | 0.002 | 1.000 | 0.004 | 1.000 | 0.003 | 1.000 | 0.003 | 1.000 | 0.003 | 1.000 | 0.003 |
| CR_LF | JAN_BL | 12 | 1.000 | 0.004 | 1.000 | 0.003 | 1.000 | 0.002 | 0.998 | 0.006 | 1.000 | 0.003 | 1.000 | 0.004 |
|  | JAN_HF | 12 | 1.000 | 0.004 | 1.000 | 0.003 | 1.000 | 0.001 | 0.928 | 0.004 | 1.000 | 0.005 | 1.000 | 0.002 |
|  | JAN_LF | 12 | 1.000 | 0.003 | 0.961 | 0.002 | 1.000 | 0.005 | 0.802 | 0.002 | 1.000 | 0.003 | 1.000 | 0.003 |
|  | TAC_BL | 12 | 1.000 | 0.005 | 1.000 | 0.003 | 1.000 | 0.001 | 0.970 | 0.001 | 1.000 | 0.002 | 1.000 | 0.005 |
|  | TAC_HF | 11 | 1.000 | 0.002 | 1.000 | 0.004 | 1.000 | 0.005 | 0.984 | 0.002 | 1.000 | 0.007 | 0.970 | 0.005 |
|  | TAC_LF | 12 | 1.000 | 0.003 | 1.000 | 0.003 | 1.000 | 0.003 | 0.961 | 0.002 | 1.000 | 0.002 | 1.000 | 0.002 |
| JAN_BL | JAN_HF | 12 | 1.000 | 0.004 | 1.000 | 0.003 | 1.000 | 0.004 | 1.000 | 0.003 | 1.000 | 0.003 | 1.000 | 0.002 |
|  | JAN_LF | 12 | 1.000 | 0.002 | 1.000 | 0.004 | 1.000 | 0.006 | 1.000 | 0.001 | 1.000 | 0.004 | 1.000 | 0.002 |
|  | TAC_BL | 12 | 1.000 | 0.003 | 1.000 | 0.003 | 1.000 | 0.001 | 1.000 | 0.002 | 1.000 | 0.003 | 1.000 | 0.005 |
|  | TAC_HF | 11 | 1.000 | 0.003 | 1.000 | 0.002 | 1.000 | 0.003 | 1.000 | 0.005 | 1.000 | 0.001 | 1.000 | 0.002 |
|  | TAC_LF | 12 | 1.000 | 0.004 | 1.000 | 0.003 | 1.000 | 0.003 | 1.000 | 0.002 | 1.000 | 0.004 | 1.000 | 0.003 |
| JAN_HF | JAN_LF | 12 | 0.980 | 0.003 | 0.967 | 0.004 | 0.990 | 0.002 | 0.961 | 0.001 | 0.990 | 0.003 | 1.000 | 0.004 |
|  | TAC_BL | 12 | 1.000 | 0.002 | 1.000 | 0.002 | 1.000 | 0.001 | 1.000 | 0.002 | 1.000 | 0.005 | 1.000 | 0.002 |
|  | TAC_HF | 11 | 1.000 | 0.001 | 1.000 | 0.005 | 1.000 | 0.002 | 1.000 | 0.002 | 1.000 | 0.002 | 0.980 | 0.002 |
|  | TAC_LF | 12 | 1.000 | 0.003 | 1.000 | 0.004 | 1.000 | 0.005 | 1.000 | 0.004 | 1.000 | 0.003 | 1.000 | 0.004 |
| JAN_LF | TAC_BL | 12 | 1.000 | 0.005 | 1.000 | 0.002 | 1.000 | 0.004 | 0.996 | 0.002 | 1.000 | 0.003 | 1.000 | 0.004 |
|  | TAC_HF | 11 | 1.000 | 0.001 | 1.000 | 0.003 | 1.000 | 0.001 | 1.000 | 0.004 | 1.000 | 0.004 | 1.000 | 0.002 |
|  | TAC_LF | 12 | 1.000 | 0.002 | 1.000 | 0.002 | 1.000 | 0.002 | 1.000 | 0.004 | 1.000 | 0.005 | 1.000 | 0.001 |
| TAC_BL | TAC_HF | 11 | 1.000 | 0.004 | 1.000 | 0.001 | 1.000 | 0.002 | 0.973 | 0.003 | 1.000 | 0.006 | 1.000 | 0.002 |
|  | TAC_LF | 12 | 1.000 | 0.004 | 1.000 | 0.006 | 1.000 | 0.002 | 0.959 | 0.003 | 1.000 | 0.003 | 1.000 | 0.006 |
| TAC_HF | TAC_LF | 11 | 0.880 | 0.006 | 0.963 | 0.005 | 0.970 | 0.003 | 0.824 | 0.005 | 0.990 | 0.003 | 0.570 | 0.001 |


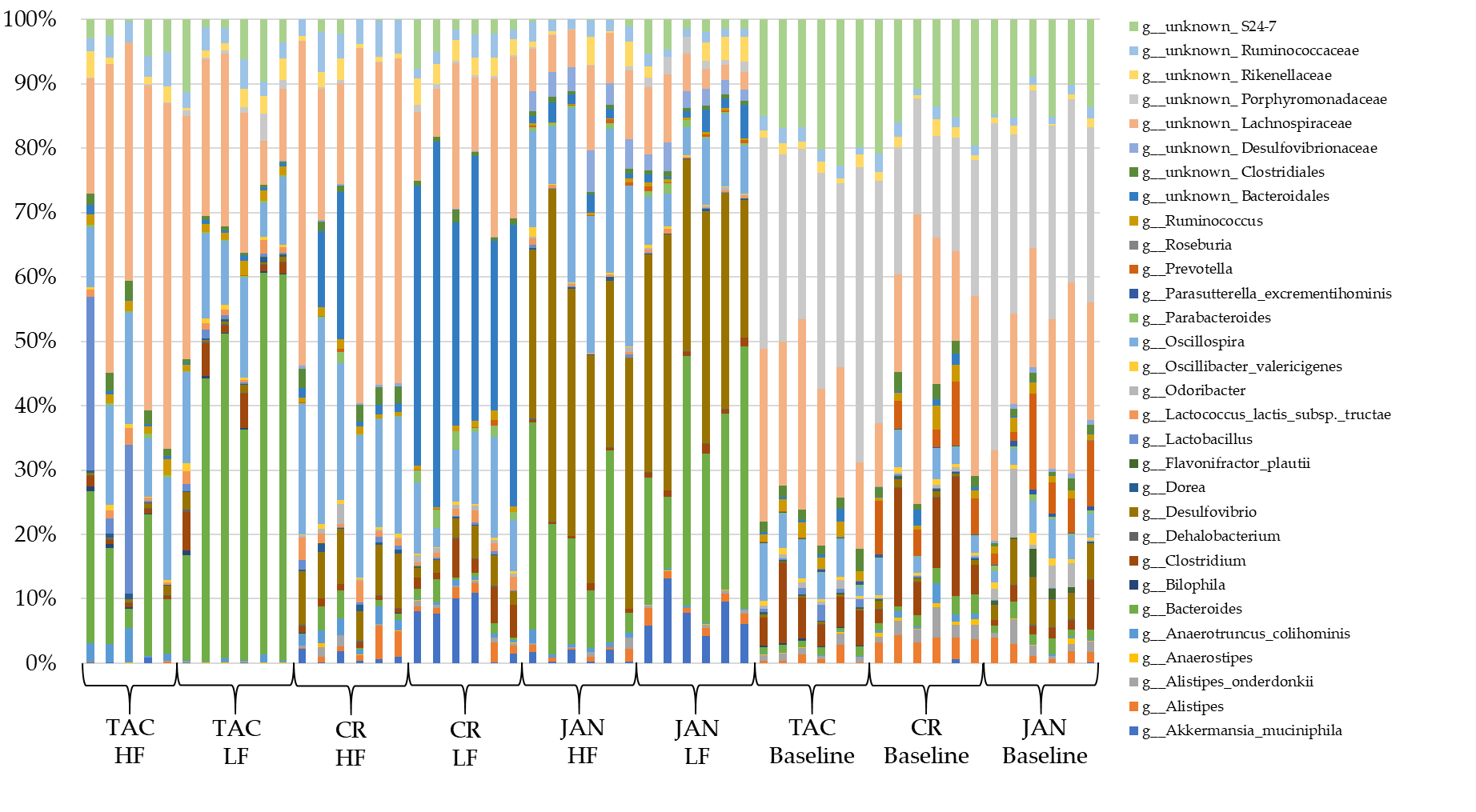


**Figure S4.** Bacterial taxonomy on genus level of all individual cecum samples. A threshold of the relative abundance was set to 0.25%. HF = high-fat diet, LF = low-fat diet. TAC = Taconic, CR = Charles River, JAN = Janvier.


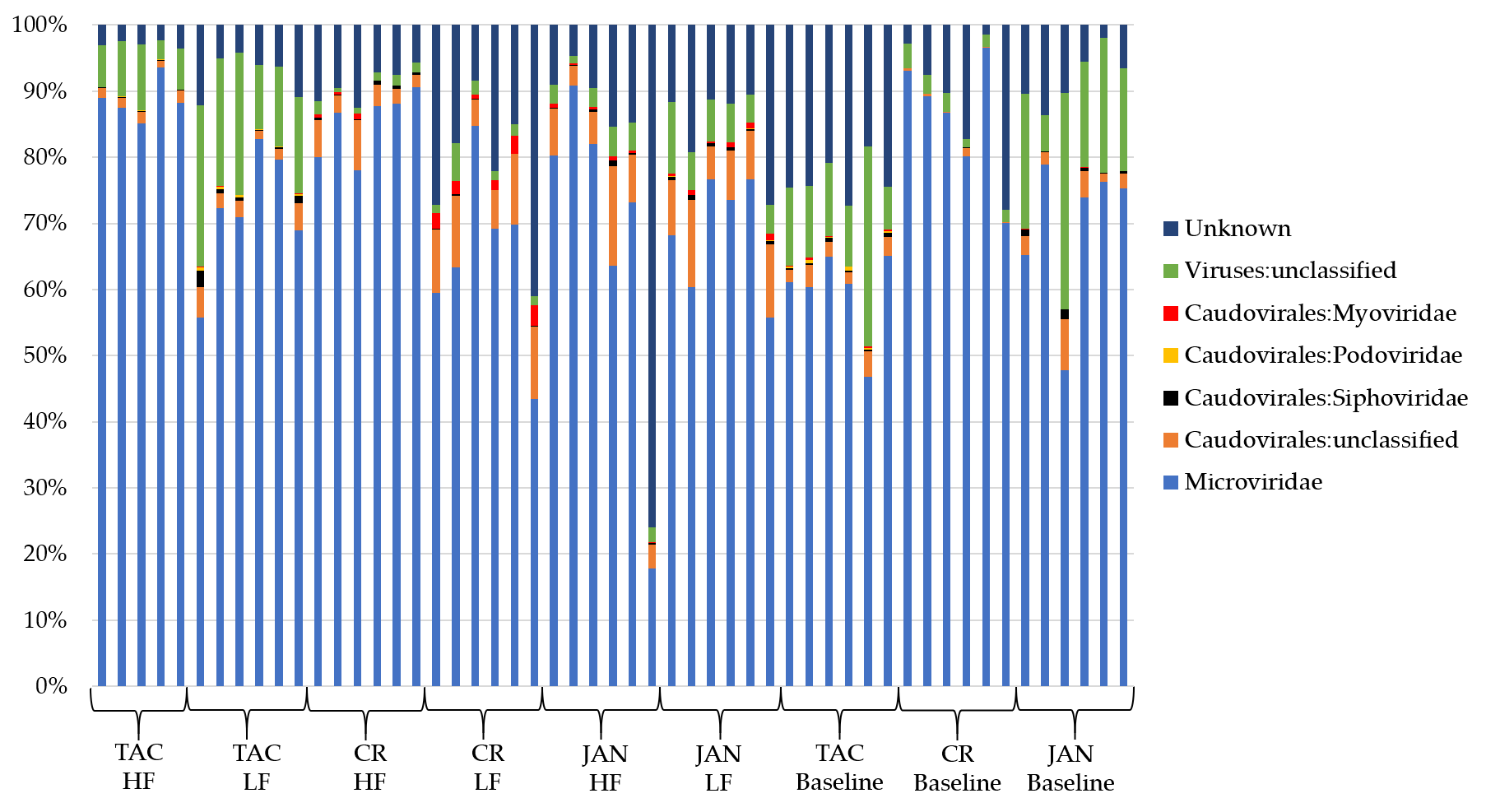


**Figure S5.** Viral taxonomy on family level of all individual cecum samples. A threshold of the relative abundance was set to 0.25%. HF = high-fat diet, LF = low-fat diet. TAC = Taconic, CR = Charles River, JAN = Janvier.


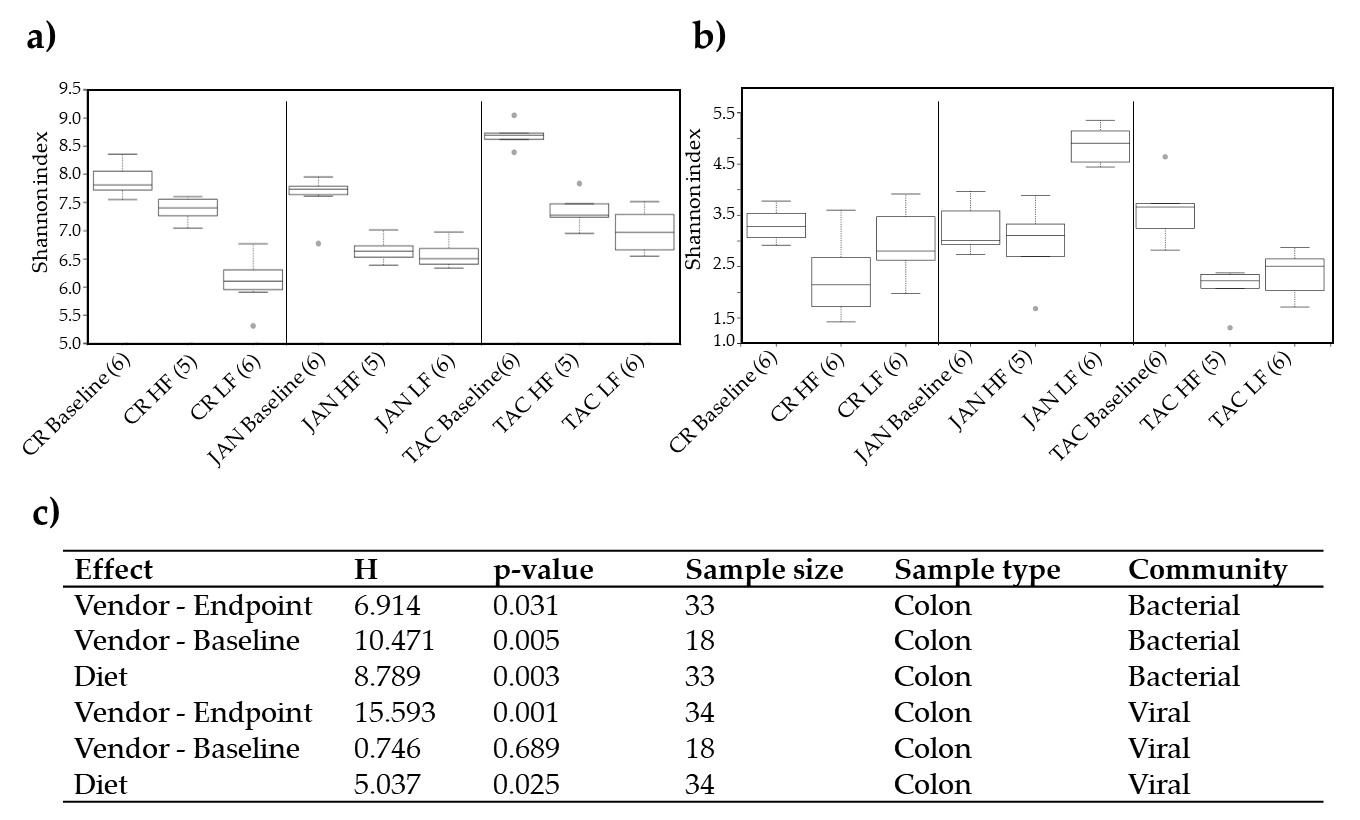


**Figure S6.** Shannon index of the colon a) bacterial and b) viral community at baseline (5 weeks of age) and after 13 weeks on low -fat or high -diet (18 weeks of age), respectively. The parentheses show the number of samples from each group included in the plot and grey dots indicate outliers. c) Kruskal Wallis group analysis of the Shannon diversity index of the effects of diet and vendor at baseline and endpoint. LF = low-fat diet, HF = high-fat diet, CR = Charles River, JAN = Janvier, TAC = Taconic.


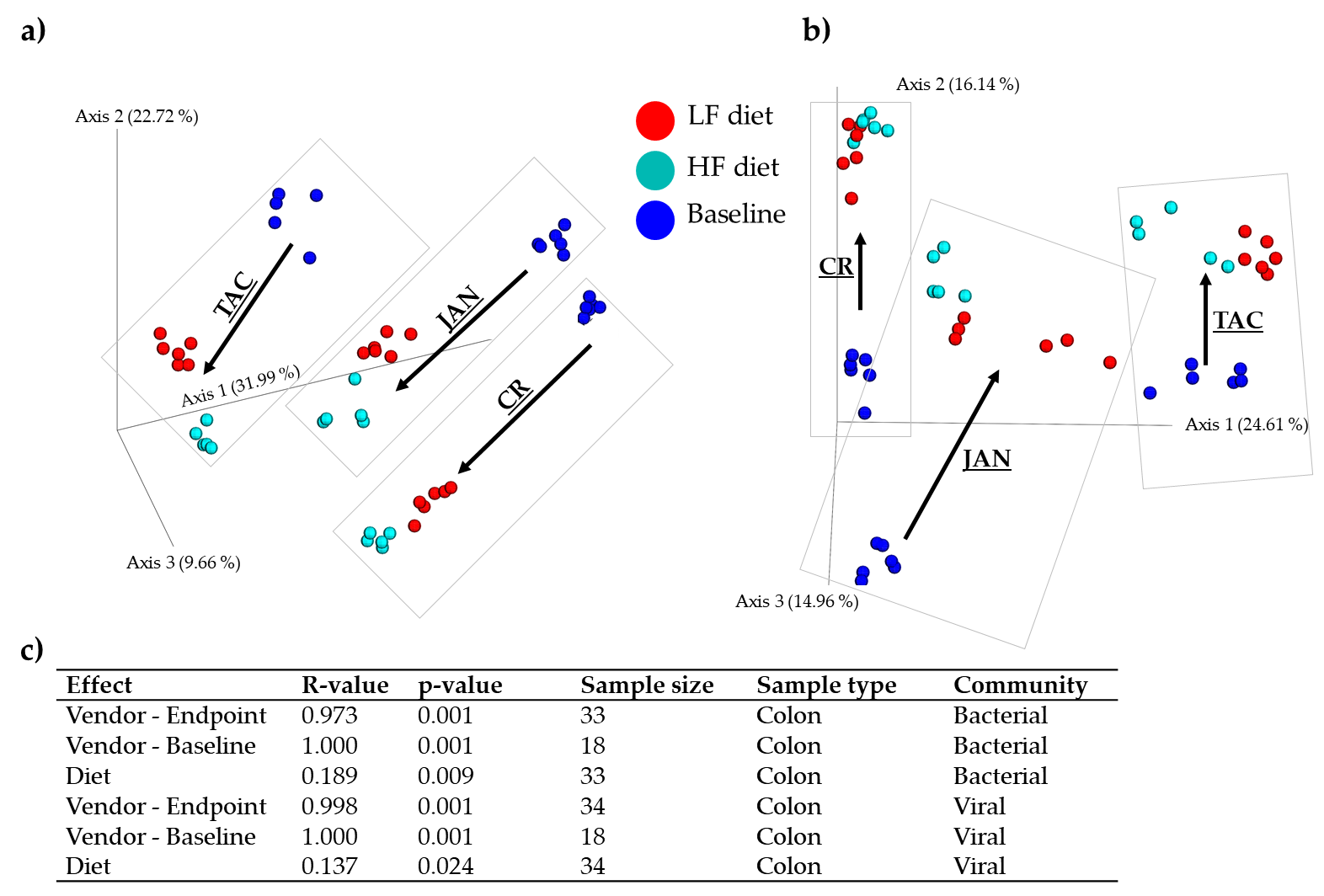


**Figure S7.** Bray Curtis dissimilarity metric PCoA based plots of a) the colon bacterial community and b) viral community at baseline (5 weeks of age) and at endpoint after 13 weeks on low- fat or high- diet (18 weeks of age), respectively. c) ANOSIM of the Bray Curtis distances of the effects of diet and vendor at baseline and endpoint. Grey boxes frame the samples associated to the mice vendor. LF = low-fat diet, HF = high-fat diet, CR = Charles River, JAN = Janvier, TAC = Taconic


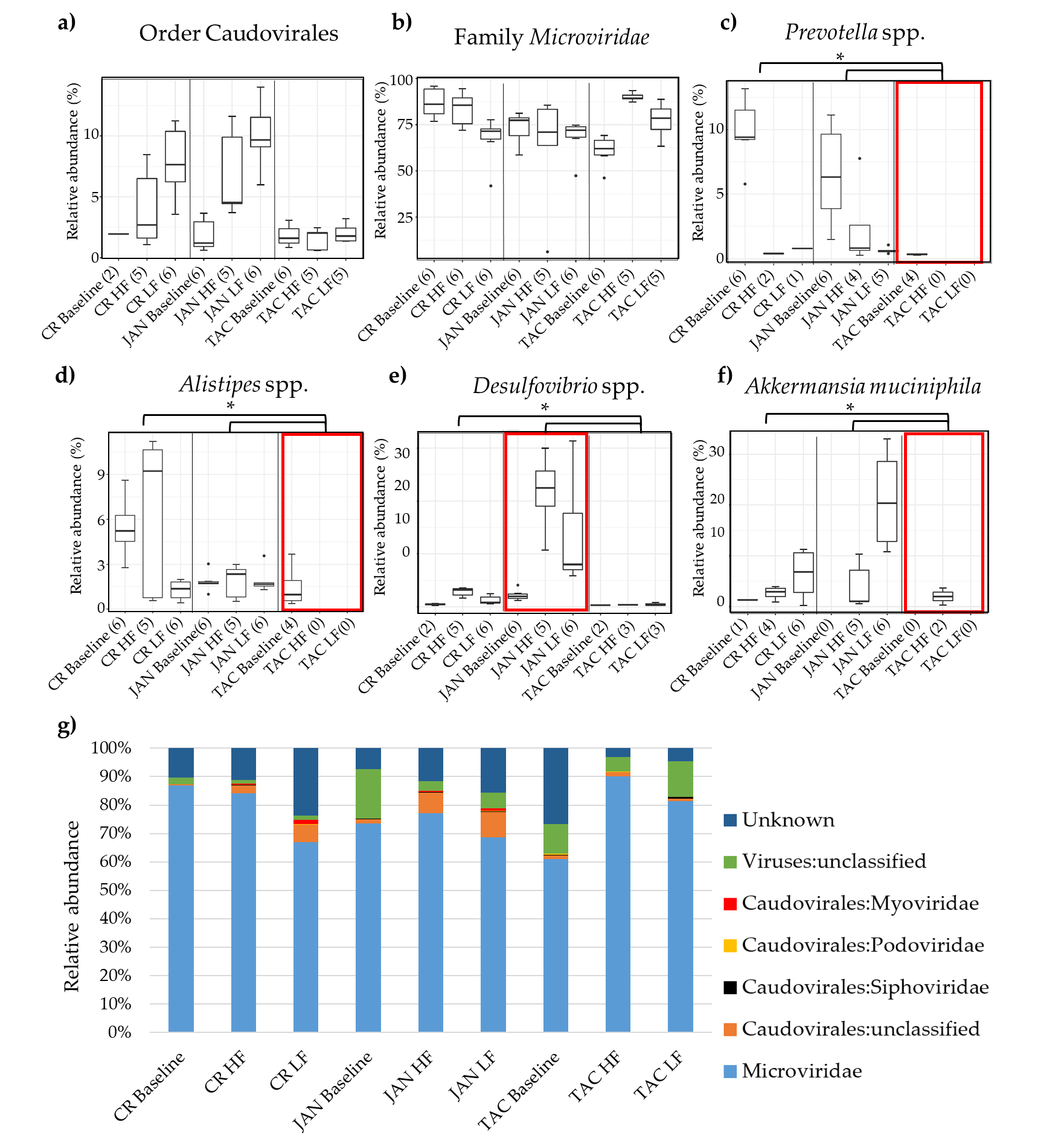


**Figure S8.** Relative abundance of a) the order Caudovirales and b) the family Microviridae. Differences in the relative abundance of Prevotella spp., Alistipes spp., Desulfovibrio spp., and Akkermansia muciniphila between vendors and diet are illustrated in respectively c), d), e), and f). The most abundant viral taxonomies are illustrated by bar plots in g). The parentheses show the number of samples from each group included in the plot. Black dots indicate outliers and the red boxes mark the vendor with interesting differences in bacterial abundance. Black branches and stars mark the significant bacterial differences in abundance between vendors, * = p < 0.05, based on pairwise Wilcoxon rank sum test. LF = low-fat diet, HF = high-fat diet, CR = Charles River, JAN = Janvier, TAC = Taconic.


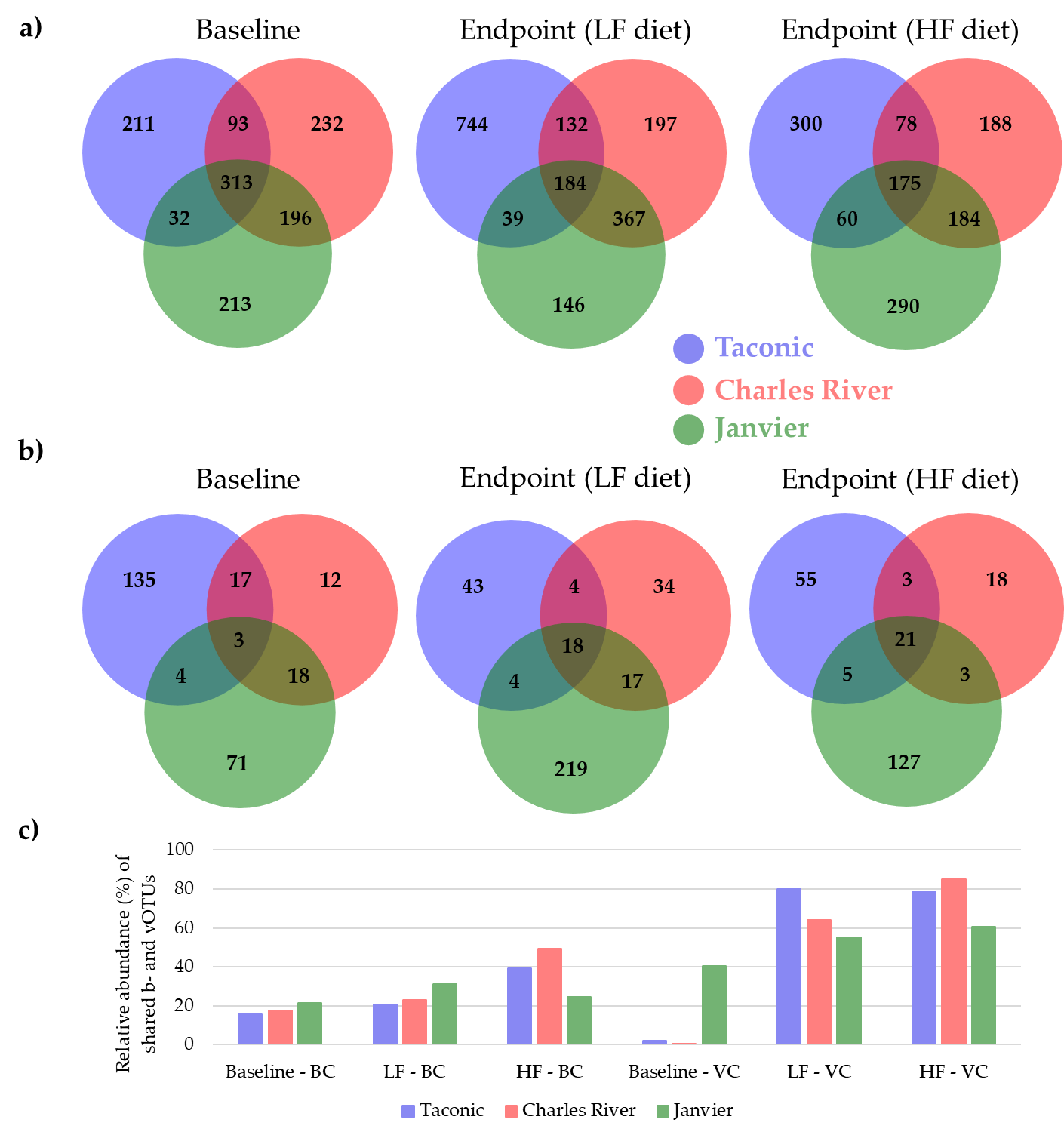


**Figure S9.** Venn diagrams illustrating the number of shared colon a) bacterial and b) viral OTUs (b- and vOTU’s) amongst mice purchased from three vendors at baseline (5 weeks old) and endpoint (18 weeks old) on either high-fat (HF) or low-fat (LF) diet. The numbers inside the Venn diagram indicate the amount of shared OTUs. c) Bar plot illustrating the sum of the relative abundance of the shared b- and vOTUs from baseline to endpoint. BC = Bacterial community, VC = viral community.


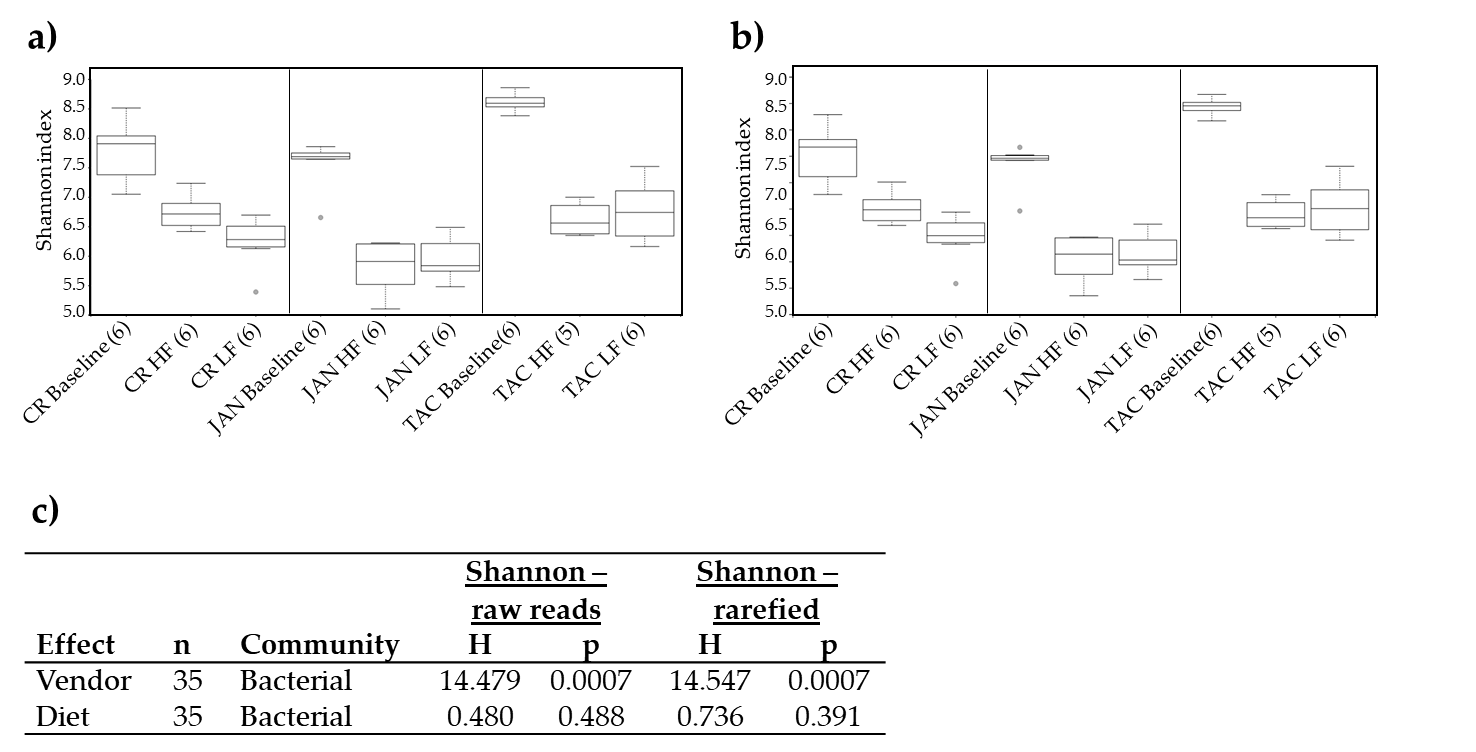


**Figure S10.** Box and whiskers plot comparing the bacterial α-diversity (Shannon index) when the analysis was based on either raw read count a) or rarefied read counts b). (c) Kruskal Wallis analysis emphasising that rarefaction was not necessary in this case.


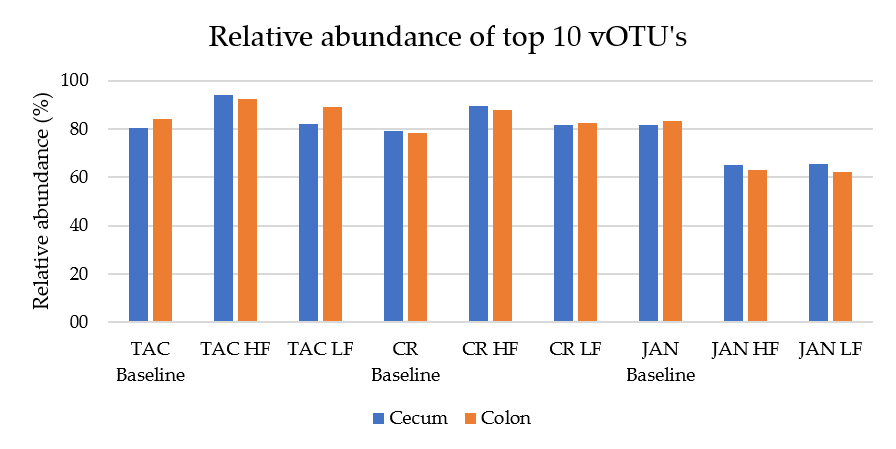


**Figure S11.** Bar plot of the mean relative abundance (%) represented by the top 10 vOTUs covering all groups of diet and vendor of both cecum and colon samples.


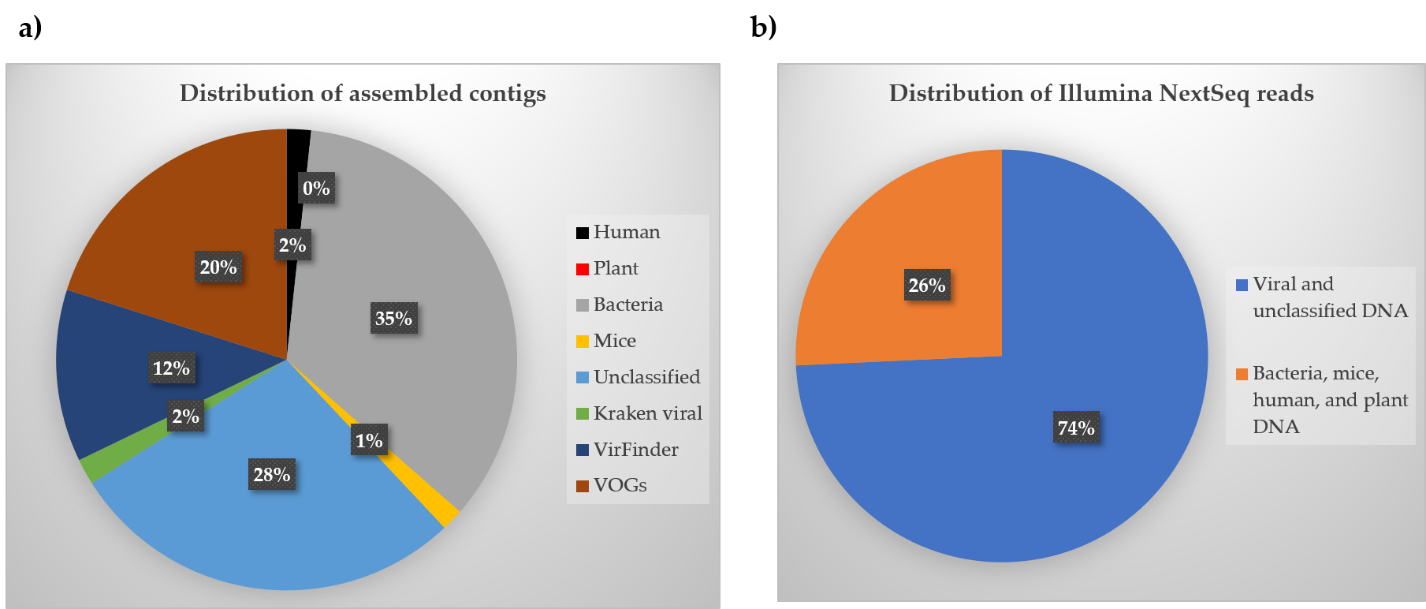


**Figure S12.** Pie plots showing the distribution of the assembled contigs based on origin a) and Illumina NextSeq reads b) of the sequenced metaviromes. Contigs identified by VOGs, Kraken, VirFinder and unclassified DNA constituted the vOTU-table whereas the residuals were removed as contaminations. More than 74% of the Illumina NextSeq reads were used for viral and unclassified contigs (vOTUs).


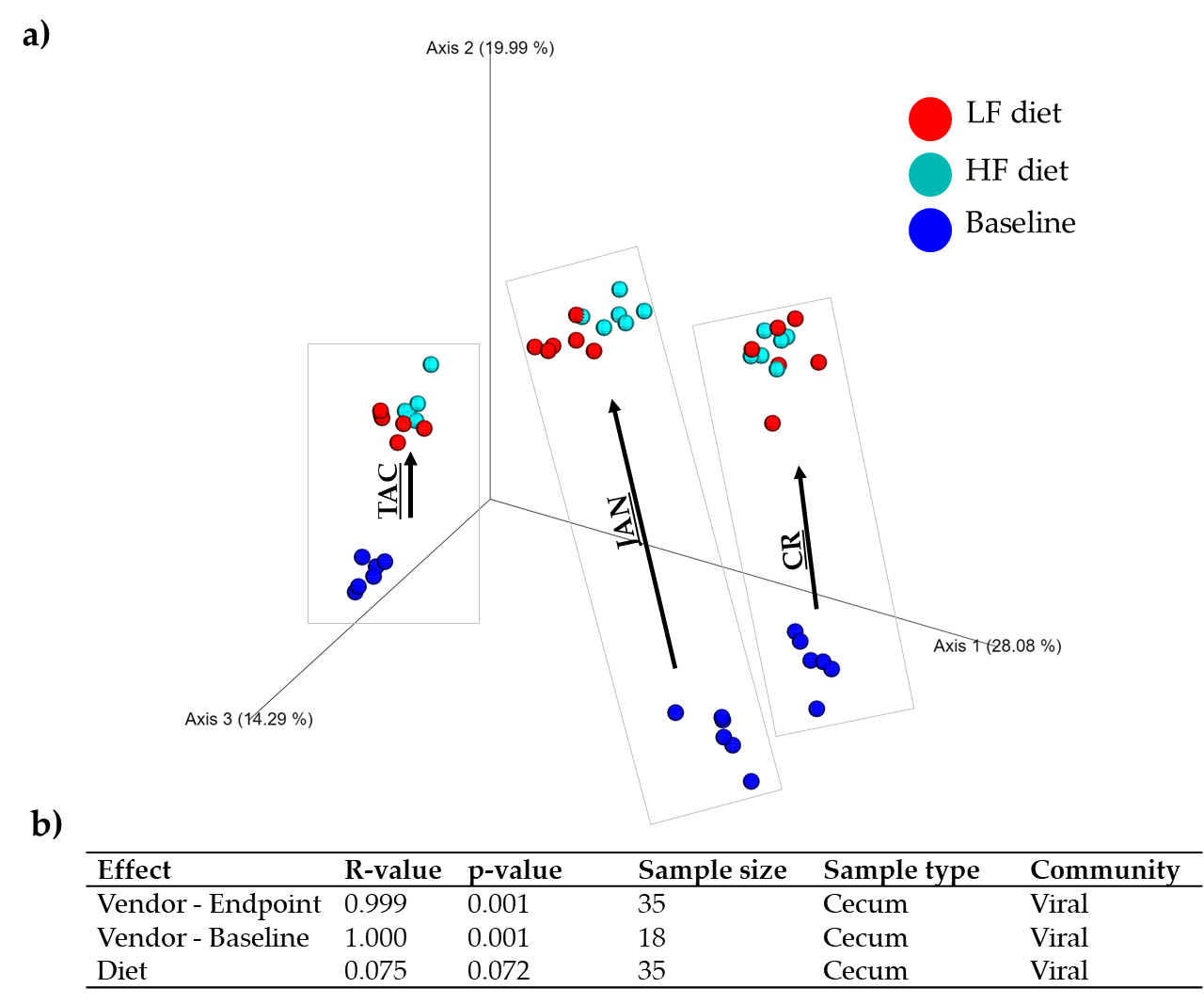


**Figure S13.** Bray Curtis dissimilarity metric PCoA based plots of the caecal a) viral community at baseline (5 weeks of age) and at endpoint after 13 weeks on low-fat or high-diet (18 weeks of age), respectively. The PCoA plot only includes vOTUs larger than 3,000 bp and with no detectable integrase genes. b) ANOSIM of the Bray Curtis distances of the effects of diet and vendor at baseline and endpoint. Gray boxes frame the samples associated to the mice vendor. CR = Charles River, JAN = Janvier, TAC = Taconic.
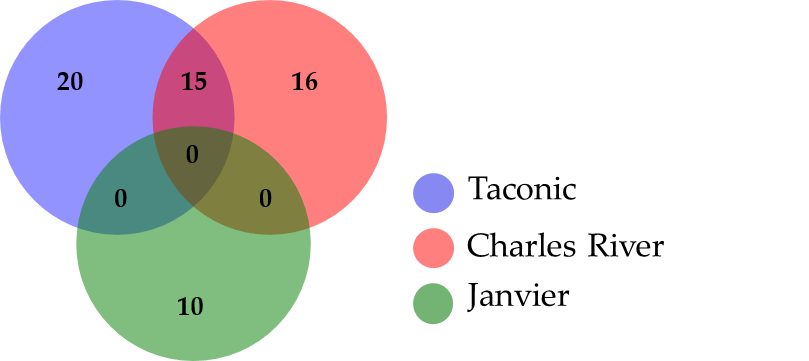


**Figure S14.** Venn diagram showing the amount vOTUs in “core-viromes” independently of diet and time of all eighteen mice from each vendor.

**Table S4.** The table list the taxonomy of the shared b- and vOTU’s showed in Figure 5a & 5b for cecum samples and Figure S14a & S14b for colon samples. LF = low-fat diet, HF = high-fat diet, CR = Charles River, JAN = Janvier, TAC = Taconic, BC = bacterial community, VC = viral community.

| **Taxonomy of shared b- and vOTU’s - Cecum** | | | | | |
| --- | --- | --- | --- | --- | --- |
| **Baseline - BC** | **LF - BC** | **HF - BC** | **Baseline - VC** | **LF - VC** | **HF - VC** |
| *Clostridium spp.* | *Akkermansia muciniphila* | *Anaerotruncus colihominis* | Unclassified Caudovirales | Microviridae | Microviridae |
| Lachnospiraceae | *Anaerotruncus spp.* | *Clostridium spp.* |  | Unclassified viruses | Unclassified viruses |
| *Alistipes spp.* | *Bacteroides spp.* | *Desulfovibrio spp.* |  | Unknown | Unknown |
| *Oscillibacter spp.* | *Clostridium spp.* | *Lactobacillus animalis* |  |  |  |
| *Oscillospira spp.* | Lachnospiraceae | *Parabacteroides distasonis* |  |  |  |
|  | *Lactobacillus animalis* | *Lactococcus lactis subsp.tructae* |  |  |  |
|  | *Lactococcus lactis subsp.tructae* | *Oscillospira spp.* |  |  |  |
|  | Mogibacteriaceae | Lachnospiraceae |  |  |  |
|  | *Oscillibacter spp.* | Ruminococcaceae |  |  |  |
|  | *Oscillospira spp.* |  |  |  |  |
|  | *Parabacteroides distasonis* |  |  |  |  |
|  | *Ruminococcus spp.* |  |  |  |  |
| **Taxonomy of shared b- and vOTU’s - Colon** | | | | | |
| *Alistipes spp.* | *Akkermansia muciniphila* | *Akkermansia muciniphila* | Unclassified Caudovirales | Microviridae | Microviridae |
| *Clostridium* spp*.* | *Anaerotruncus* spp*.* | *Anaerotruncus* spp*.* |  | Unclassified viruses | Phycodnaviridae |
| Lachnospiraceae | *Clostridium* spp*.* | *Dorea* spp*.* |  | Unknown | Unclassified viruses |
| *Lactobacillus animalis* | Lachnospiraceae | Lachnospiraceae |  |  | Unknown |
| *Oscillibacter* spp. | *Lactobacillus animalis* | *Lactobacillus animalis* |  |  |  |
|  | *Lactococcus lactis subsp.tructae* | *Lactococcus lactis subsp.tructae* |  |  |  |
|  | Ruminococcaceae | Ruminococcaceae |  |  |  |
|  | *Oscillospira* spp*.* | Rikenellaceae |  |  |  |
|  | *Parabacteroides distasonis* | *Oscillibacter* spp*.* |  |  |  |
|  | *Ruminococcus* spp*.* | *Oscillospira* spp*.* |  |  |  |
|  |  | *Parabacteroides distasonis* |  |  |  |
|  |  | *Ruminococcus* spp*.* |  |  |  |

**Table S5.** PERMANOVA test was performed to highlight that the impact of vendor exceeded the impact of diet on the bacterial and viral gut microbiota composition.

| **Viral community** | **Df** | **SumOfSqs** | **R2** | **F** | **Pr(>F)** |
| --- | --- | --- | --- | --- | --- |
| Vendor - Endpoint | 2 | 1.92 | 0.58 | 29.01 | 0.001 |
| Diet - Endpoint | 1 | 0.36 | 0.11 | 10.98 | 0.001 |
| Residual | 31 | 1.02 | 0.31 | NA | NA |
| Total | 34 | 3.30 | 1.00 | NA | NA |
| **Bacterial community** |  |  |  |  |  |
| Vendor - Endpoint | 2 | 4.10 | 0.50 | 19.31 | 0.001 |
| Diet - Endpoint | 1 | 0.73 | 0.09 | 6.86 | 0.001 |
| Residual | 31 | 3.29 | 0.41 | NA | NA |
| Total | 34 | 8.12 | 1.00 | NA | NA |

**Table S6.** List of bacterial genera and/or species found to significantly differ between vendors. The p-values are based on Wilcoxon rank sum test.

| **Bacterial taxonomy** | **p-value** |
| --- | --- |
| *Desulfovibrio* spp. | 0.0000 |
| *Lactobacillus animalis* | 0.0001 |
| *Lactococcus lactis* spp. | 0.0030 |
| *Parabacteroides distasonis* | 0.0003 |
| *Prevotella* spp. | 0.0001 |
| *Ruminococcus* spp. | 0.0000 |
| *Clostridium aerotolerans* | 0.0413 |
| *Bacteroides* spp. | 0.0061 |
| *Anaerotruncus colihominis* | 0.0025 |
| *Alistipes* spp*.* | 0.0129 |
| *Akkermansia muciniphila* | 0.0004 |
