# Supplementary figures and images for: Mouse Vendor Influence on the Bacterial and Viral Gut Composition Exceeds the Effect of Diet
Source: Viruses. 2019 May 13;11(5):435. doi: 10.3390/v11050435 (PMC6563299; doi:10.3390/v11050435)

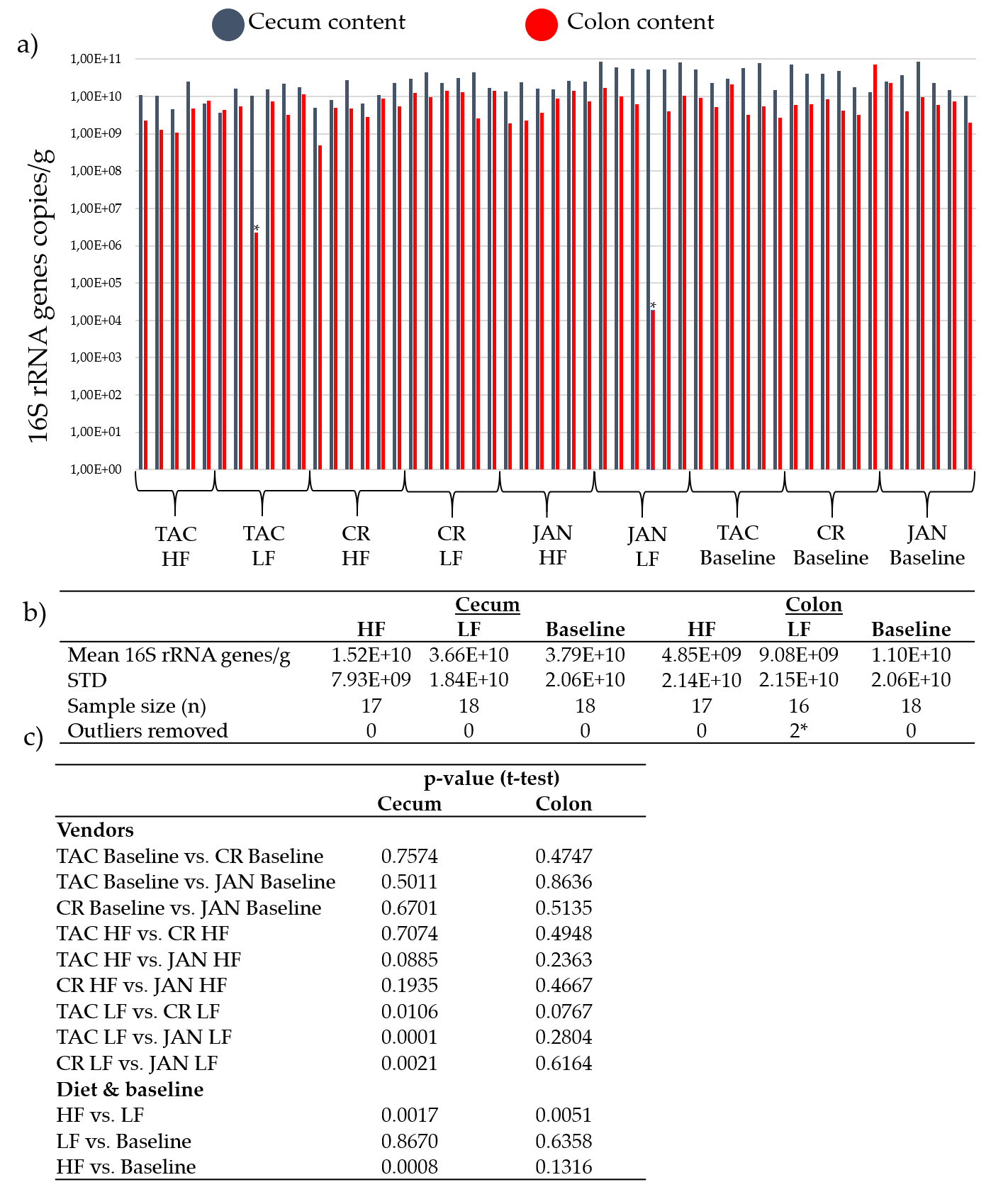

Supplement: Supplementary file 1 [file viruses-11-00435-s001.zip › Supplemental materials_revised/Supplemental materials/Figure S1.tif]

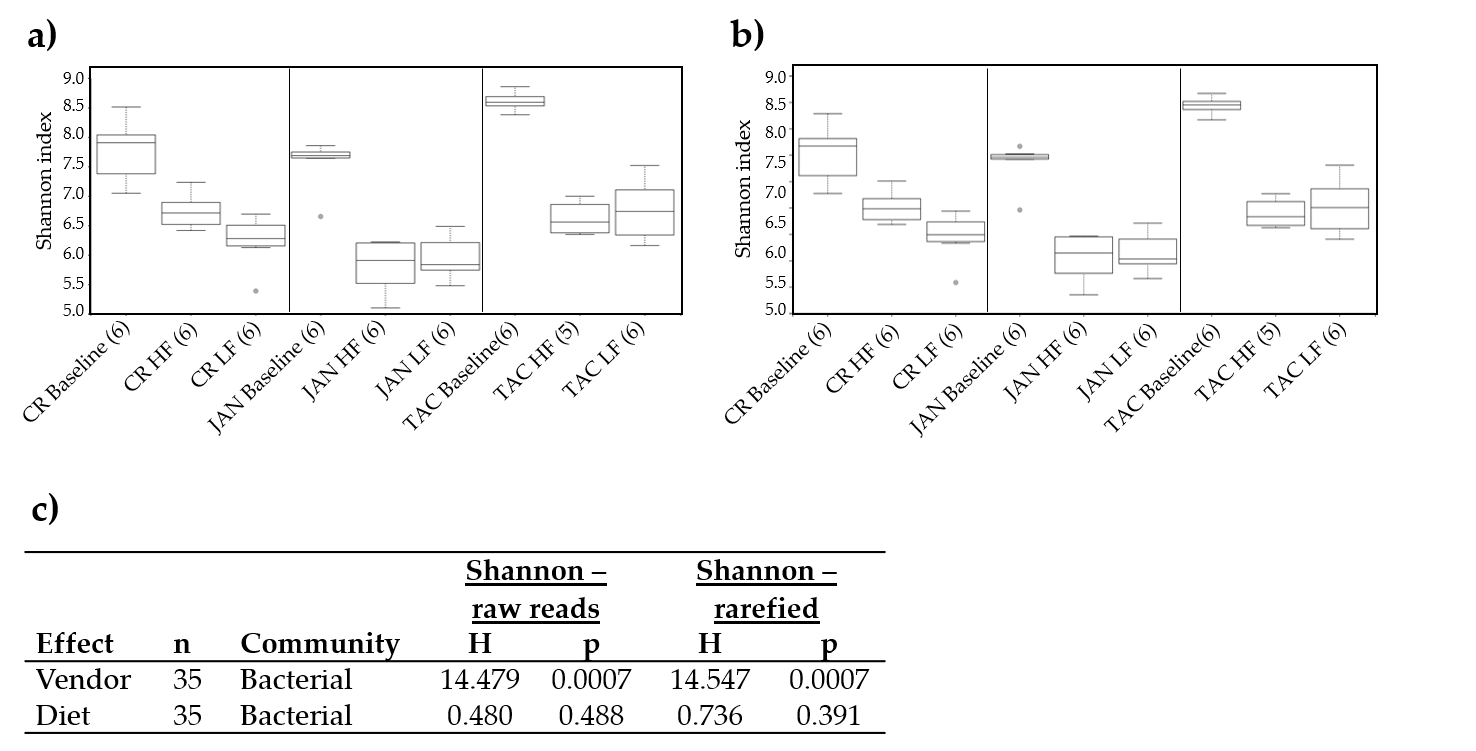

Supplement: Supplementary file 1 [file viruses-11-00435-s001.zip › Supplemental materials_revised/Supplemental materials/Figure S10.tif]

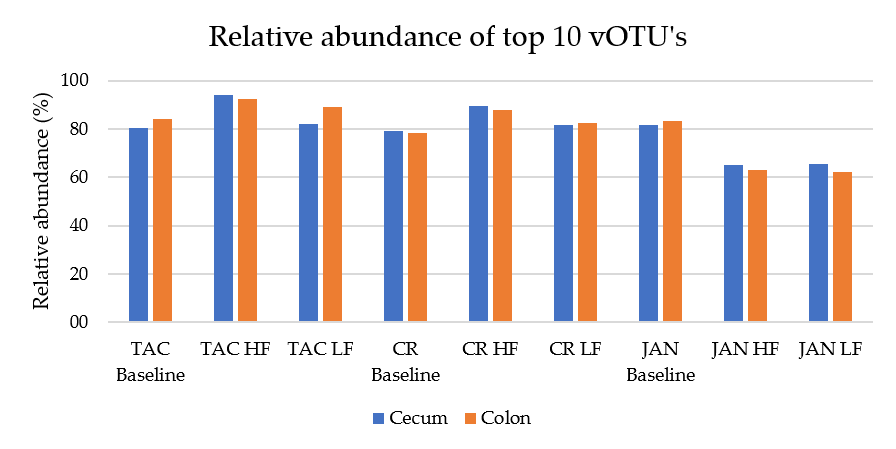

Supplement: Supplementary file 1 [file viruses-11-00435-s001.zip › Supplemental materials_revised/Supplemental materials/Figure S11.tif]

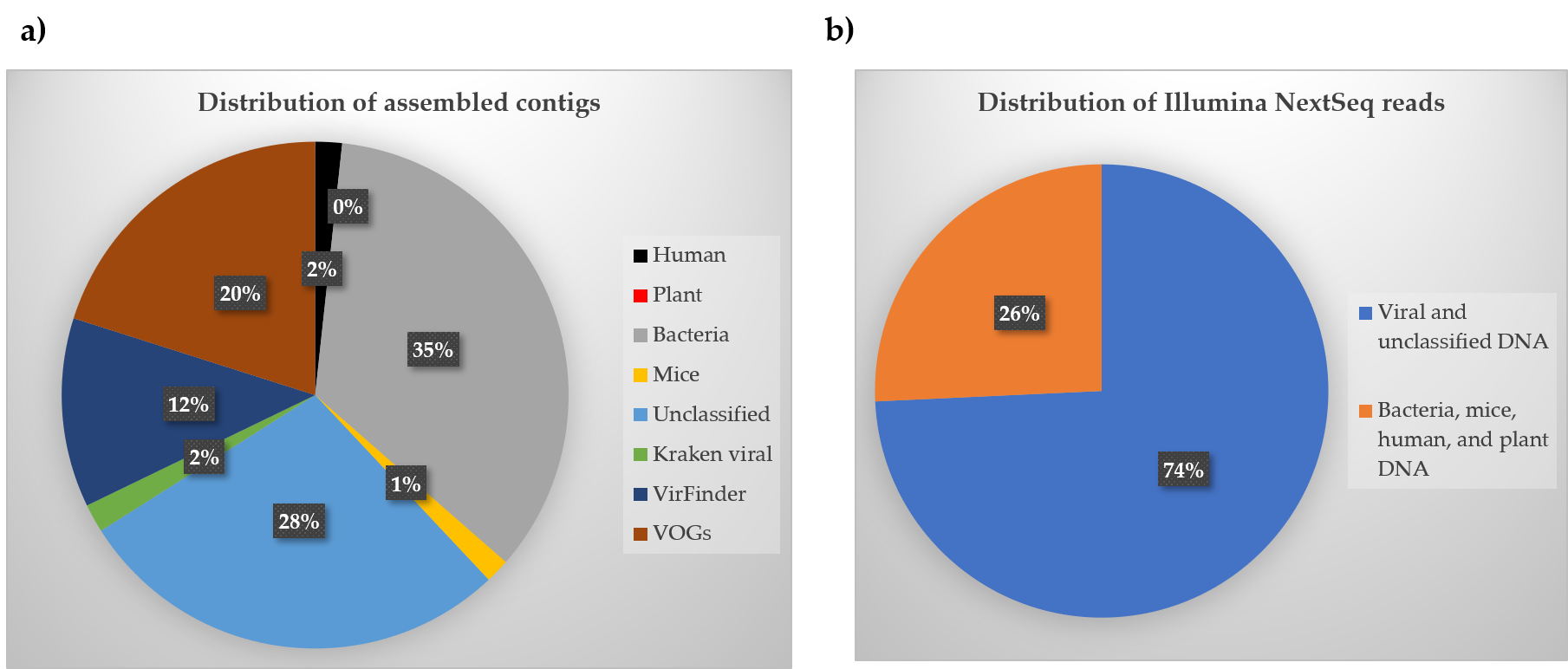

Supplement: Supplementary file 1 [file viruses-11-00435-s001.zip › Supplemental materials_revised/Supplemental materials/Figure S12.tif]

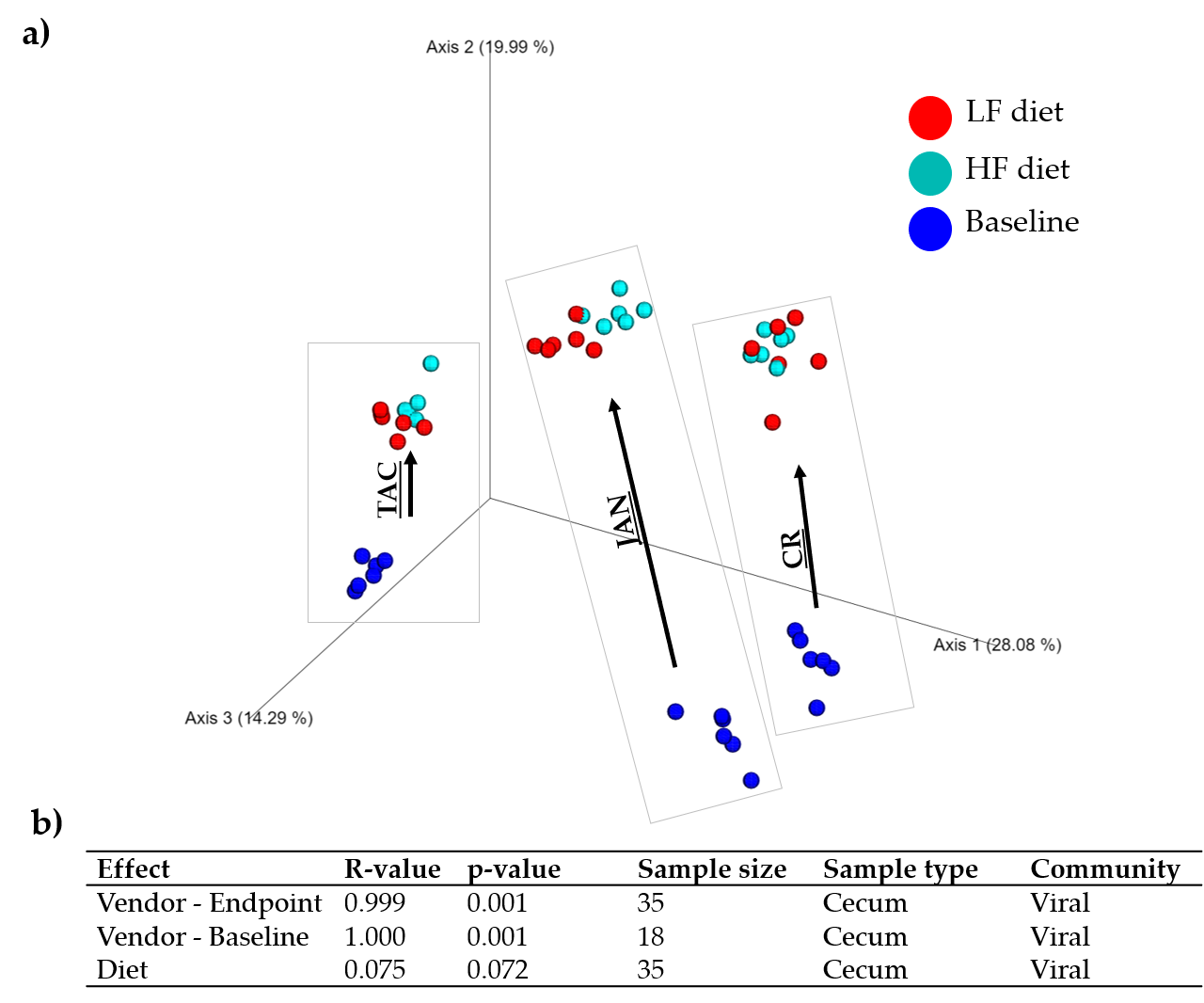

Supplement: Supplementary file 1 [file viruses-11-00435-s001.zip › Supplemental materials_revised/Supplemental materials/Figure S13.tif]

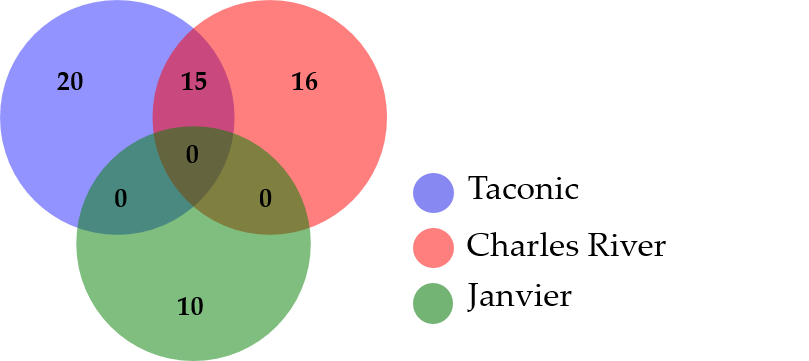

Supplement: Supplementary file 1 [file viruses-11-00435-s001.zip › Supplemental materials_revised/Supplemental materials/Figure S14.tif]

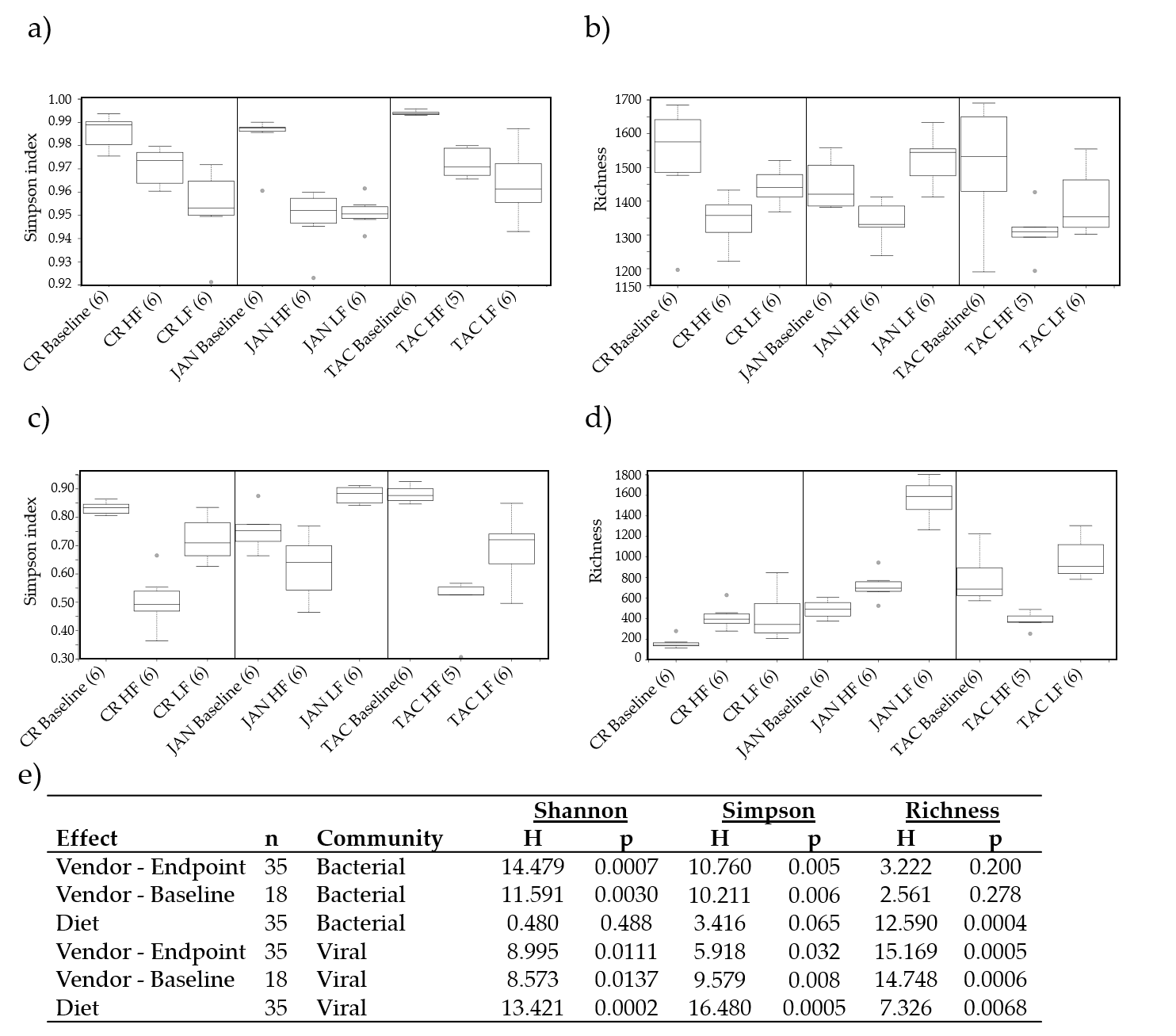

Supplement: Supplementary file 1 [file viruses-11-00435-s001.zip › Supplemental materials_revised/Supplemental materials/Figure S2.tif]

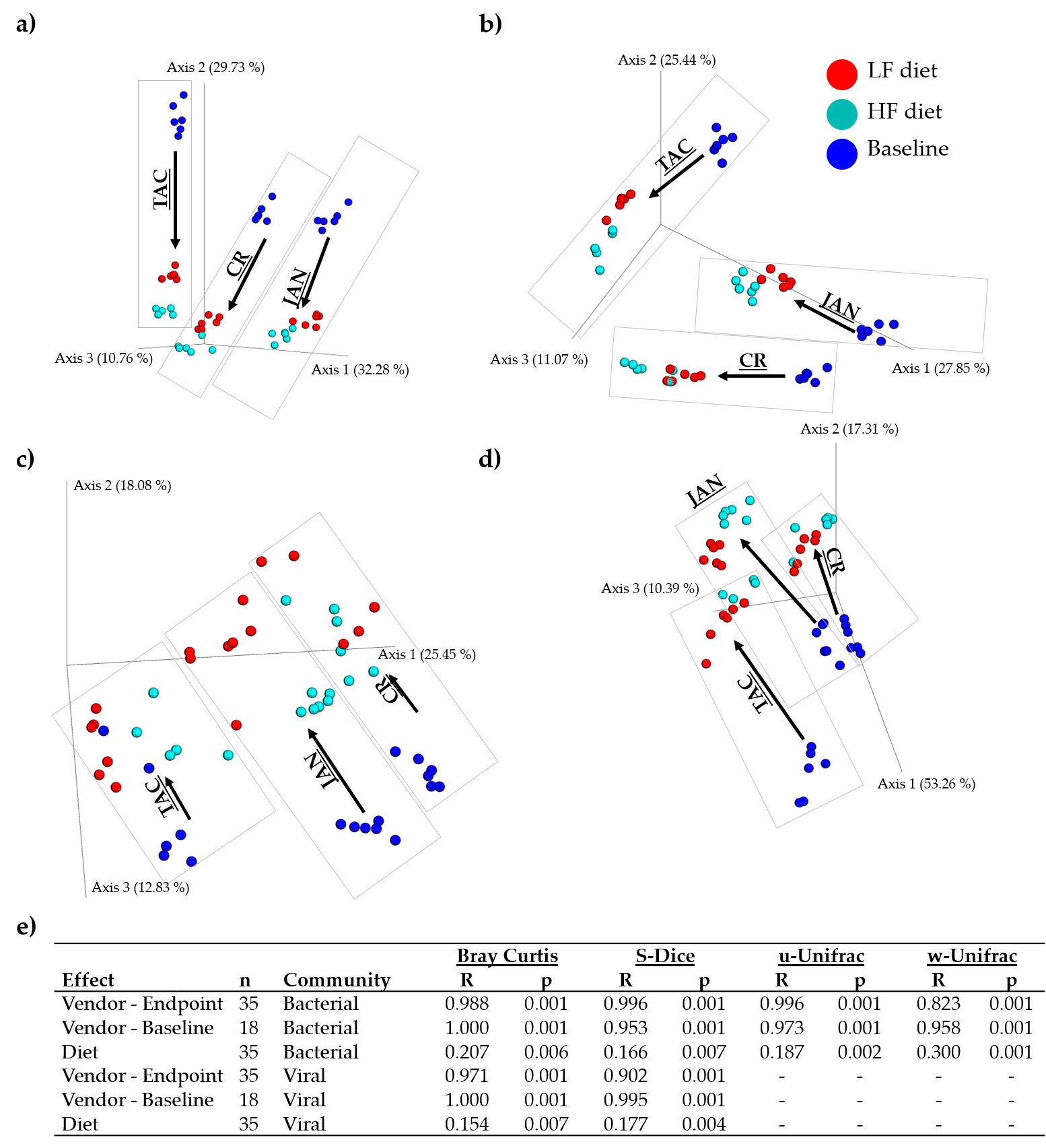

Supplement: Supplementary file 1 [file viruses-11-00435-s001.zip › Supplemental materials_revised/Supplemental materials/Figure S3.tif]

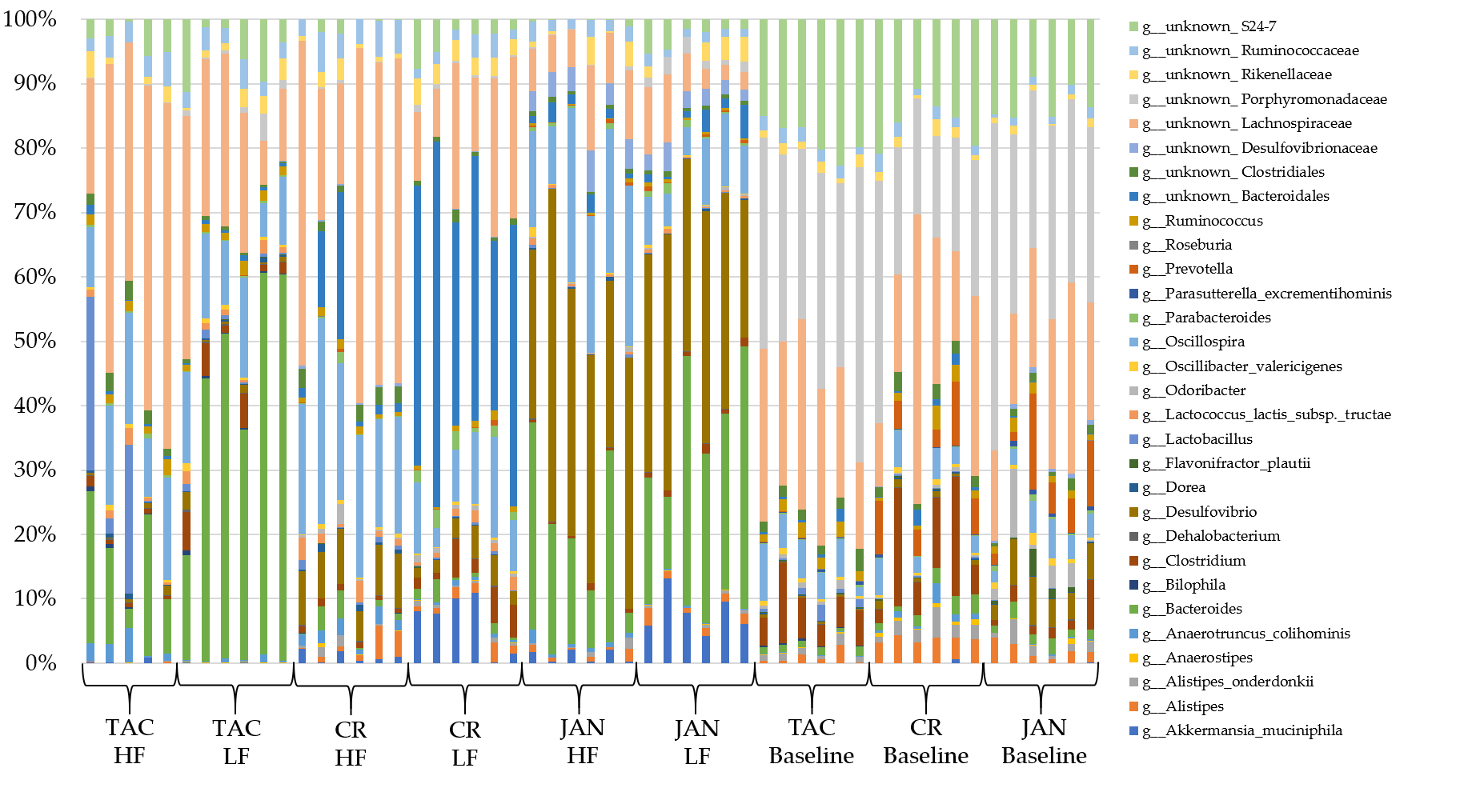

Supplement: Supplementary file 1 [file viruses-11-00435-s001.zip › Supplemental materials_revised/Supplemental materials/Figure S4.tif]

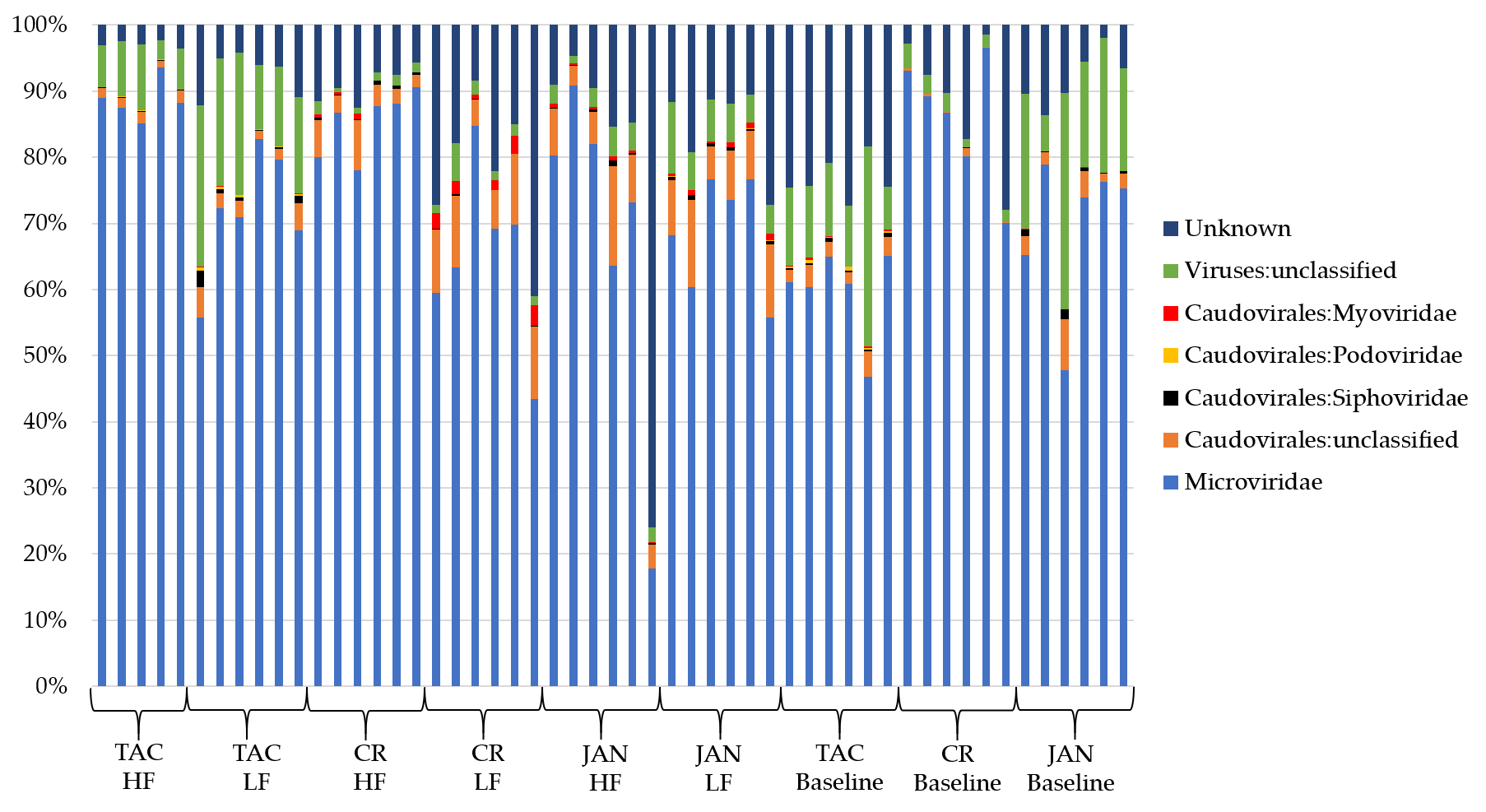

Supplement: Supplementary file 1 [file viruses-11-00435-s001.zip › Supplemental materials_revised/Supplemental materials/Figure S5.tif]

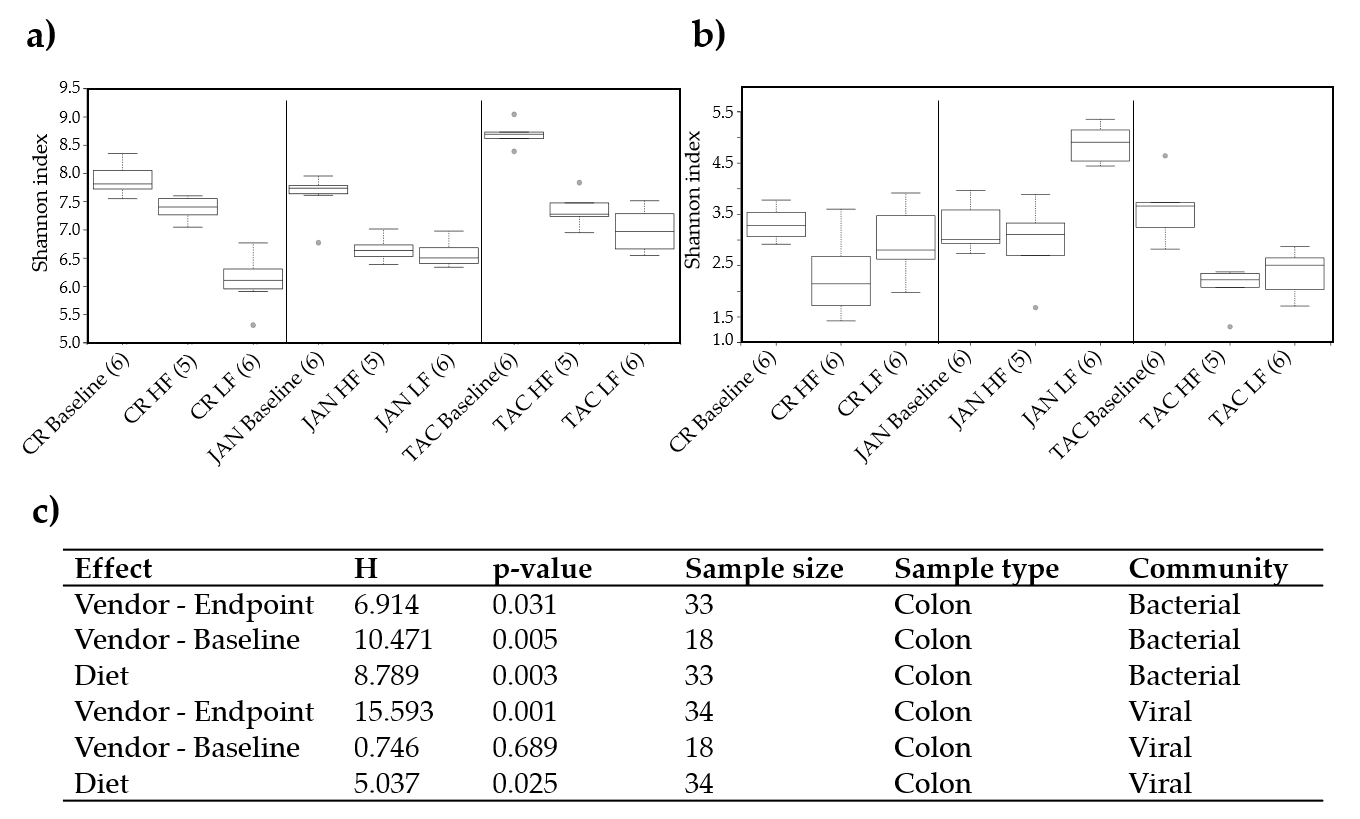

Supplement: Supplementary file 1 [file viruses-11-00435-s001.zip › Supplemental materials_revised/Supplemental materials/Figure S6.tif]

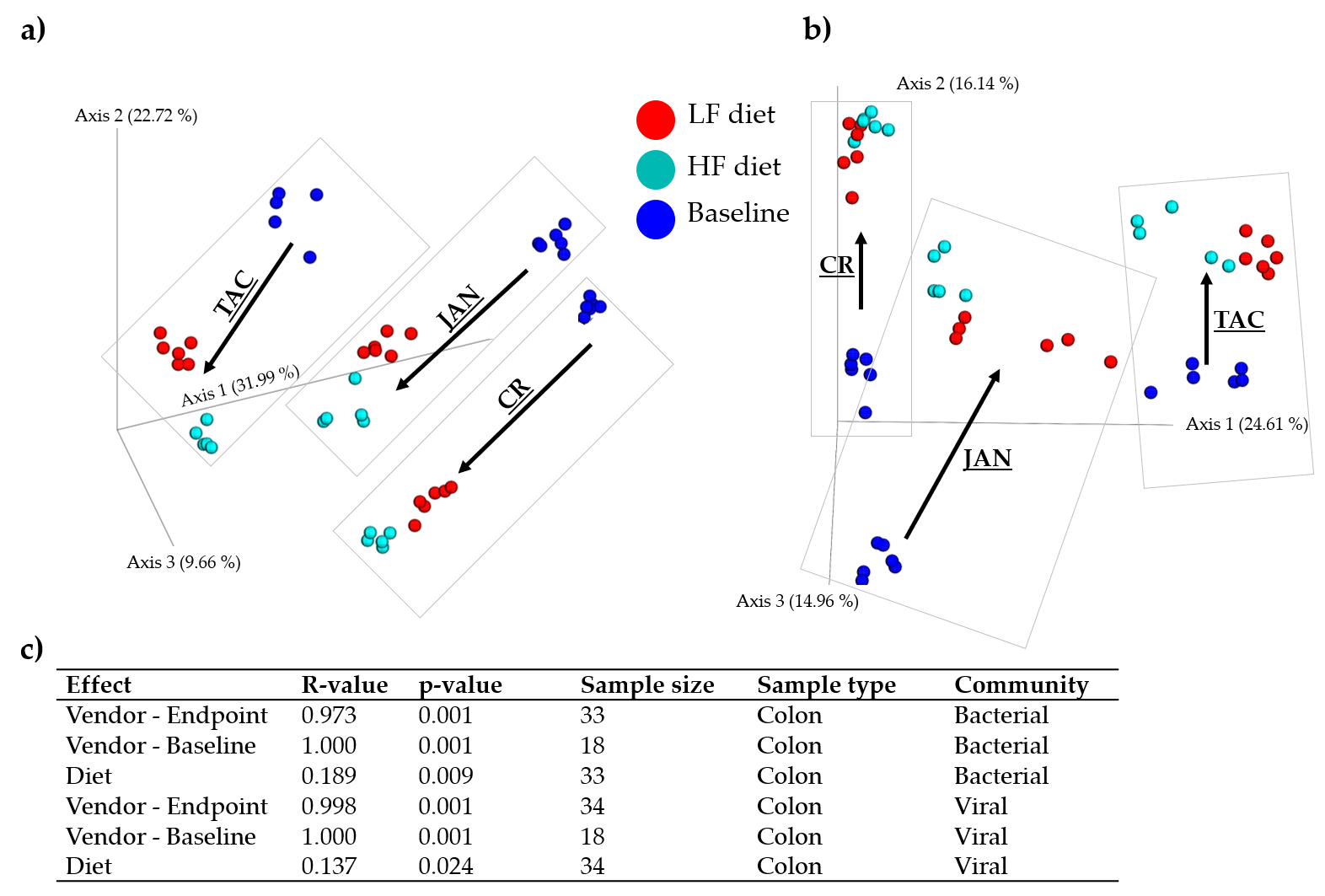

Supplement: Supplementary file 1 [file viruses-11-00435-s001.zip › Supplemental materials_revised/Supplemental materials/Figure S7.tif]

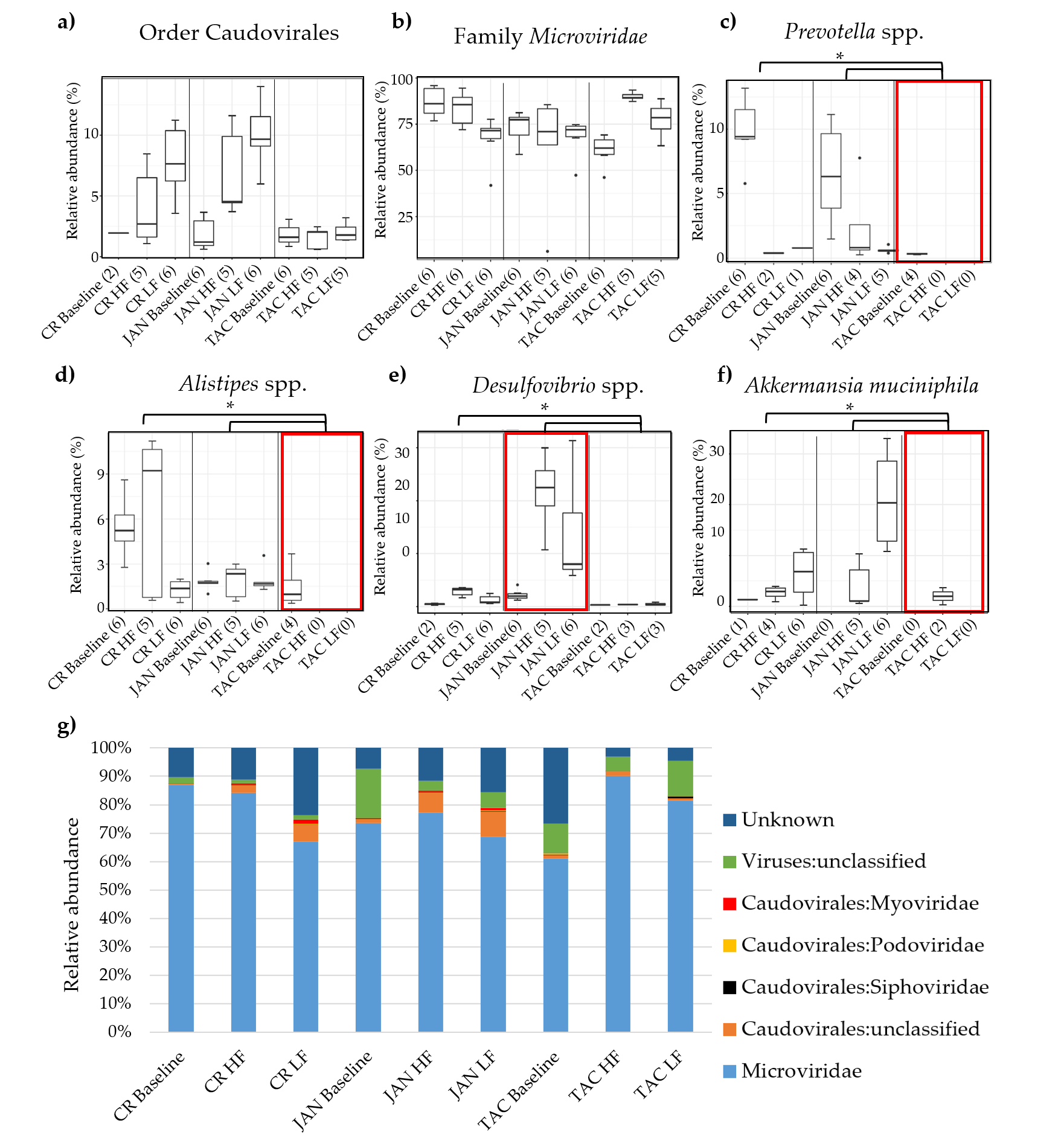

Supplement: Supplementary file 1 [file viruses-11-00435-s001.zip › Supplemental materials_revised/Supplemental materials/Figure S8.tif]

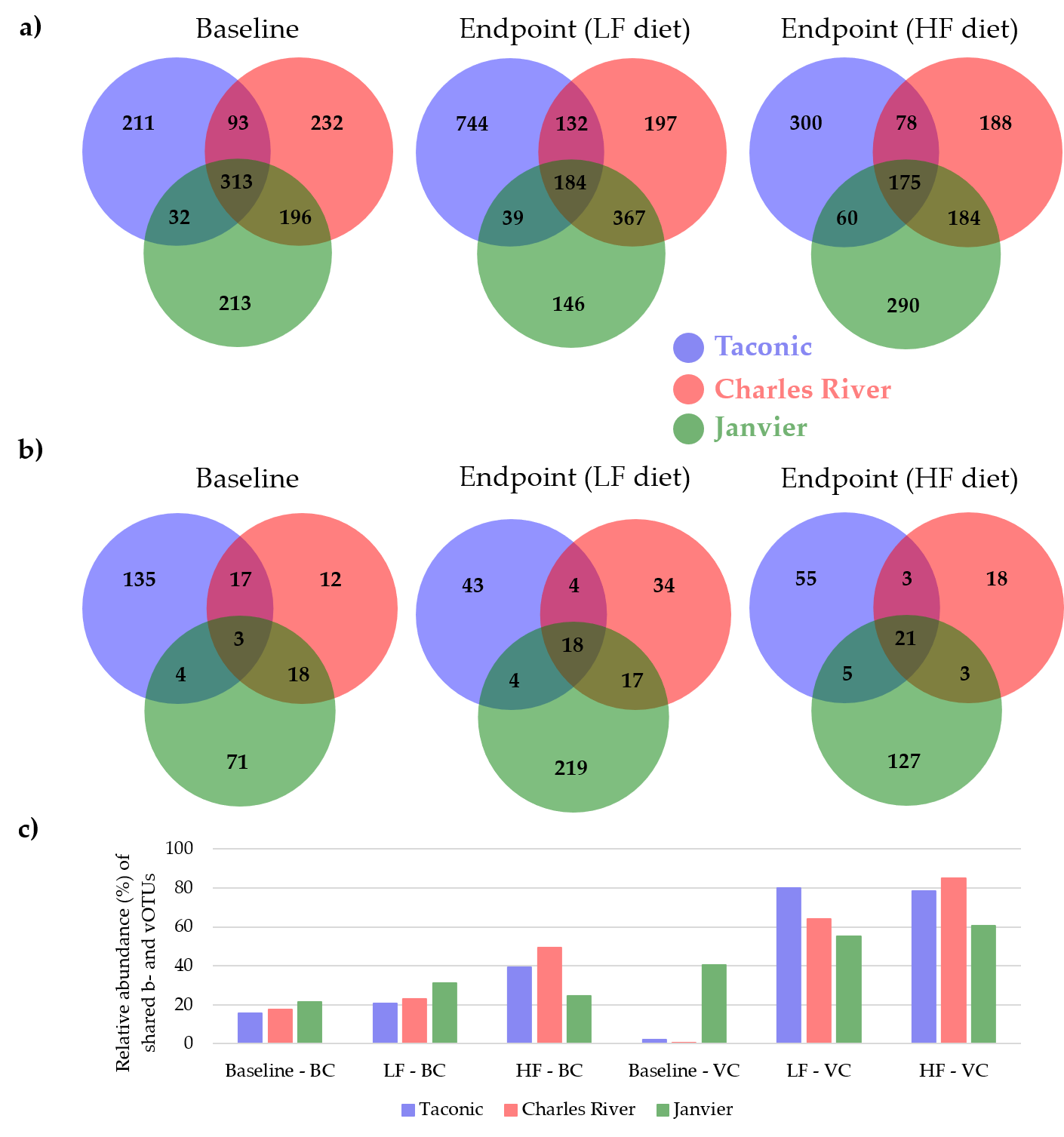

Supplement: Supplementary file 1 [file viruses-11-00435-s001.zip › Supplemental materials_revised/Supplemental materials/Figure S9.tif]
